# *Drosophila* hedgehog signaling range and robustness depend on direct and sustained heparan sulfate interactions

**DOI:** 10.3389/fmolb.2023.1130064

**Published:** 2023-02-22

**Authors:** Dominique Manikowski, Georg Steffes, Jurij Froese, Sebastian Exner, Kristina Ehring, Fabian Gude, Daniele Di Iorio, Seraphine V. Wegner, Kay Grobe

**Affiliations:** ^1^ Institute of Physiological Chemistry and Pathobiochemistry, University of Münster, Münster, Germany; ^2^ Institute of Neuro- and Behavioral Biology, University of Münster, Münster, Germany

**Keywords:** hedgehog, morphogen, *Drosophila*, wing disc, heparan sulfate, QCM-D, heparin, development

## Abstract

Morphogens determine cellular differentiation in many developing tissues in a concentration dependent manner. As a central model for gradient formation during animal development, Hedgehog (Hh) morphogens spread away from their source to direct growth and pattern formation in the *Drosophila* wing disc. Although heparan sulfate (HS) expression in the disc is essential for this process, it is not known whether HS regulates Hh signaling and spread in a direct or in an indirect manner. To answer this question, we systematically screened two composite Hh binding areas for HS *in vitro* and expressed mutated proteins in the *Drosophila* wing disc. We found that selectively impaired HS binding of the second site reduced Hh signaling close to the source and caused striking wing mispatterning phenotypes more distant from the source. These observations suggest that HS constrains Hh to the wing disc epithelium in a direct manner, and that interfering with this constriction converts Hh into freely diffusing forms with altered signaling ranges and impaired gradient robustness.

## 1 Introduction

Pattern formation in multicellular organisms involves the differential specification of cell fate by a number of highly conserved signaling pathways, including the Hedgehog (Hh) pathway (Ingham and McMahon, 2001). Upon Hh secretion to the plasma membrane of the producing cell, the 12-pass transmembrane protein Dispatched (Burke et al., 1999) releases the morphogen as a prerequisite for its spreading and signaling to distant cells that express the receptor Patched (Ptc) (Murone et al., 1999). It is currently thought that extracellular Hh spread results in a concentration gradient to directly specify the fate of each cell along this gradient (Hooper and Scott, 2005). Yet, the mode of morphogen spread from producing to receiving cells, which is essential for graded morphogen action, is not clear. Among the various possible modes by which morphogen spread to target cells may be achieved, the most current models propose lipidated Hh transport on filopodia called cytonemes (Bischoff et al., 2013; Sanders et al., 2013), on secreted vesicles called exosomes (Gradilla et al., 2014), on lipoproteins (Panakova et al., 2005) or *via* extracellular Hh diffusion to target cells [as recently discussed in (Stapornwongkul and Vincent, 2021)]. Yet, unconstrained extracellular Hh diffusion—not only of the free protein but also if associated with exosomes or lipoproteins—is difficult to envision. This is for the reason that patterning of folded epithelia bordering fluid-filled compartments, such as in the *Drosophila melanogaster* wing disc, would cause morphogen diffusion off the plane of the epithelial cell layer into the fluid-filled compartment. This, in turn, would prevent reliable and robust gradient formation (Kornberg and Guha, 2007). In our work, we tested one possible solution to this problem, suggesting that Hh may be constricted to the epithelial surface by prolonged interactions with negatively charged heparan sulfate (HS) chains, analogous to the established constriction of DNA-binding proteins in the nucleus by interactions with the negatively charged DNA (Blainey et al., 2006; Elf et al., 2007).

Indeed, genetic evidence has firmly established that unimpaired Hh signaling depends on the expression of HS proteoglycans (HSPGs) at the cell-surface. HS chains consist of alternating glucuronic acid or iduronic acid/*N*-acetylglucosamine disaccharide units with different degrees of sulfation (Sarrazin et al., 2011). The essential role of polyanionic HS in Hh biofunction was demonstrated by similar phenotypes of embryonic null mutants for hh and for genes encoding HS-modified core proteins (Han et al., 2004b; Takeo et al., 2005; Gallet et al., 2008; Ayers et al., 2010; Williams et al., 2010) or HS biosynthetic enzymes [ttv (Bellaiche et al., 1998; The et al., 1999), brother of ttv and sister of ttv (Han et al., 2004a; Bornemann et al., 2004; Takei et al., 2004)]. Although these observations may be explainable by hypothetical indirect HS functions in the guidance of cytonemes or by Hh stabilization in the matrix (Bellaiche et al., 1998, The et al., 1999; Han et al., 2004a; Han et al., 2004b; Stapornwongkul and Vincent, 2021; Aguirre-Tamaral et al., 2022), another conceivable possibility is that Hhs interact directly with HS to constrict morphogen movement to the apical side of the epithelial surface to maximize signaling (Chan et al., 2009; Bischoff et al., 2013; Aguirre-Tamaral et al., 2022). In this study, we tested this possibility based on the hypothesis that extracellular Hh constriction to the epithelial surface by interactions with linear HS chains resembles the restriction of DNA-binding protein movement along the DNA backbone in the nucleus.

To this end, we characterized the interactions of transgenic vertebrate Sonic hedgehog (Shh) and *Drosophila* Hh with HS and heparin (a highly sulfated form of HS) *in vitro*. This confirmed that the established Cardin-Weintraub (CW) motif (Cardin and Weintraub, 1989; Rubin et al., 2002; Farshi et al., 2011) and a second site containing arginines R238 and R239 (Chang et al., 2011; Whalen et al., 2013) contribute strongly to HS binding. We then overexpressed mutant Hh transgenes lacking HS-binding amino acids of the second site in *Drosophila* eye discs and wing discs. We found that interfering with HS interactions of the second site reduced Hh signaling strength close to the Hh source, and induced distant ectopic Hh target gene expression and highly variable wing mispatterning phenotypes *in vivo*. These findings suggest that direct HS interactions are required to constrain extracellular Hh diffusion to define the Hh signaling range and to increase Hh gradient robustness.

## 2 Results

### 2.1 Extracellular Hh associates with the extracellular matrix

**FIGURE 1:**
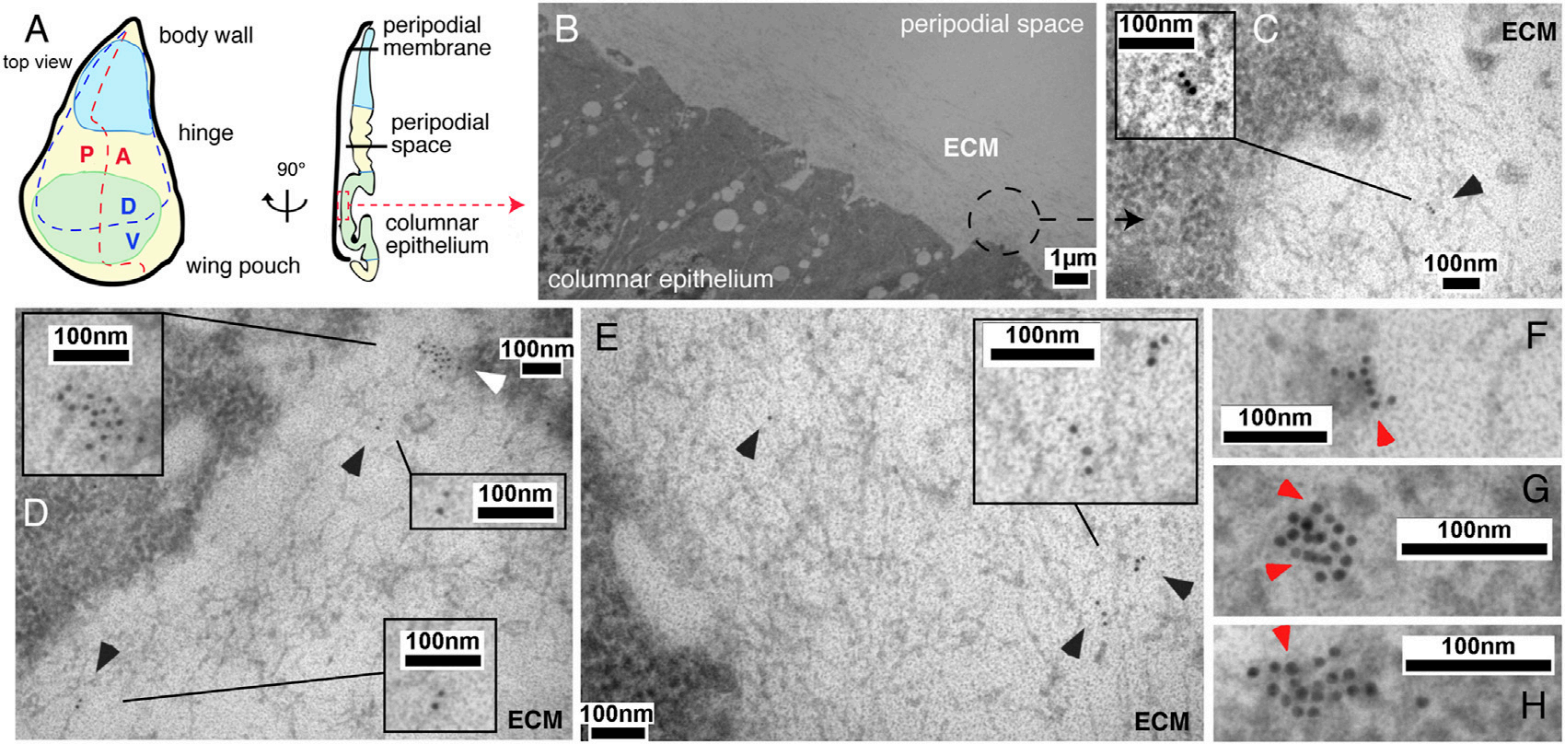
IEM analysis of Shh clusters at the wing disc surface or associated with the extracellular matrix. **(A)** Schematic of the *Drosophila* wing disc. The disc is divided along the proximal-distal axis into regions giving rise to the body wall (in blue, proximal), wing hinge (central) and wing pouch (in green, distal). This distal region contains cells of the peripodial membrane and the columnar epithelium that forms the adult wing blade. A fluid-filled peripodial space divides these cell types. Dorsal/ventral (D/V) and anterior/posterior (A/P) compartments are indicated. **(B)** Using the Gal4/UAS system, Shh was produced in the posterior wing disc compartment under engrailed (en) control. Transmission electron micrograph of the apical columnar wing disc epithelium and overlying peripodial space. ECM: fibrillar extracellular matrix meshwork. **(C–E)** Anti-Shh immunogold labeling detects untagged Shh closely associated with the fibrillar extracellular meshwork overlaying the epithelial surface (black arrowheads). Larger cell surface-associated Shh aggregates that give rise to the mobile Shh clusters were also detected [**(D)**, white arrowhead]. **(F–H)** Shh appears to be predominantly arranged non-randomly in cell surface-associated aggregates (red arrowheads).

Before their regulated release from the surface of producing cells, Hh associates with HS chains of cell surface HSPGs to form higher order Hh release platforms (Chen et al., 2004; Gallet et al., 2006; Vyas et al., 2008; Sanders et al., 2013; Ortmann et al., 2015). To determine the location and nano-architecture of these clusters, we overexpressed and visualized cell surface-associated and mobile morphogens in fly imaginal wing discs by immuno-electron microscopy (IEM). An imaginal wing disc consists of an epithelial sheet of cells that form a sac-like infolding of the epithelium in fly larvae overlayed by a fluid-filled closed compartment, called the peripodial space (Figure 1A). Hh is produced in the entire posterior epithelial layer of the wing disc compartment under the control of the transcription factor Engrailed (En) (Tabata et al., 1992; Zecca et al., 1995) and moves across the anterior/posterior (A/P) boundary into the anterior compartment to bind Ptc receptors on the same epithelial layer (Ingham et al., 1991). During this movement, Hh is thought to form a gradient of decreasing concentration with increasing distance from the A/P border. Because available antibodies failed to detect fly Hh in electron microscopy microsections, we expressed bioactive (Krauss et al., 1993; Haines and van den Heuvel, 2000) Shh in *Drosophila* wing discs using the Gal4/UAS system (Brand and Perrimon, 1993). Secreted Shh was detected in sectioned wing discs by using previously characterized anti-Shh antibodies and secondary antibodies conjugated to 10-nm gold particles (Figures 1B–H, Supplementary Figure S1) (Schürmann et al., 2018). An important observation, as shown in Figures 1C–E, is that IEM detected arrangements of non-surface-linked Shh in close association with fibrillar constituents of the extracellular matrix, in addition to the expected surface-associated Hh release platforms (Vyas et al., 2008) (Figures 1F–H, red arrowheads). This suggests that the HS-rich matrix constricts and guides the spread of solubilized Hh to the epithelial cell surface. Based on strong yet unspecific Shh interactions with *Drosophila*-derived HS, as observed *in vitro* (Supplementary Figure S1), we asked whether the observed Hh interactor at the epithelial surface consists of or contains HS.

### 2.2 Positively charged Shh amino acids interact with HS and heparin

**FIGURE 2:**
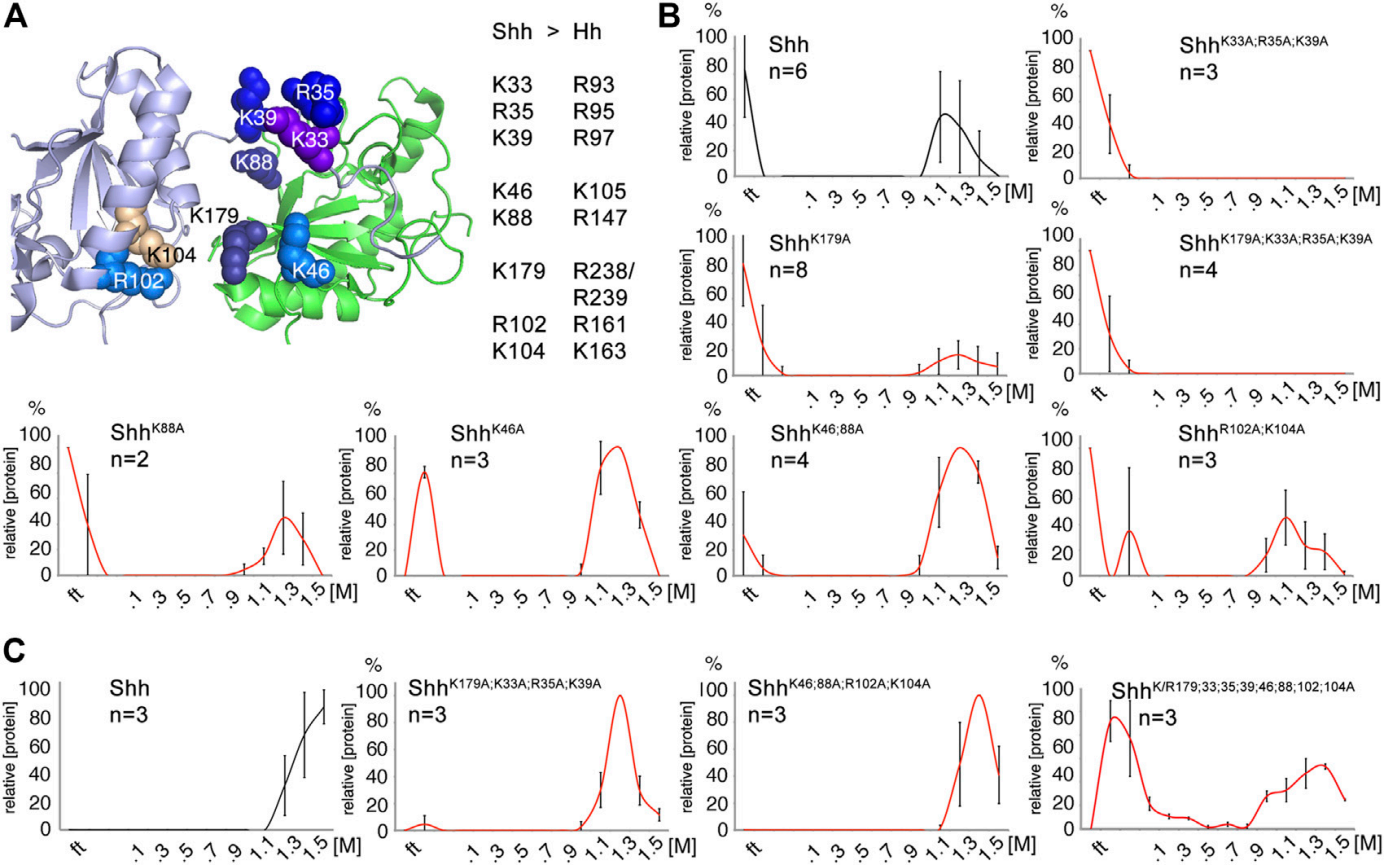
HS and heparin affinity chromatography of Shh. **(A)** Heparin-binding amino acids and their location in human Shh (PDB: 3m1n) (Pepinsky et al., 2000) are shown. The Cardin-Weintraub (CW) motif (Rubin et al., 2002) includes residues K33, R35, and K39 (Farshi et al., 2011). Residue K179 (Chang et al., 2011) and residues K88, R124, R154, and R156 (Whalen et al., 2013) are also highlighted. Residue K46 is located in close proximity to these established residues, suggesting a possible contribution to heparin binding. Residues R102 and K104 locate distant from established HS binding sites and serve as controls. **(B)** HS mixtures derived from 2 independent pools of mouse embryos were coupled to FPLC columns and Shh binding to these matrices analyzed. Shh (black line) bound to embryo-derived HS, but binding of mutant proteins (red lines) lacking CW amino acids K33, R35, and K39 was completely abolished. The relative amount of HS-binding Shh^K179A^ proteins was reduced. Note that, despite their different interaction dynamics, all proteins eluted from the HS matrix at similar salt concentrations. **(C)** Additional basic residues contribute to the binding of all Shh variants to highly sulfated heparin. Unlike for HS, protein association to heparin is fast, as indicated by absence of proteins in the flow-through (ft), but proteins progressively appear in the flow-through as more of the positively-charged residues were mutated (bottom right). The number of independently conducted experiments and standard deviations between the experiments are shown in the graphs. Black line: Shh, red lines: Shh variant proteins. See Table S1 for exact statistical results.

Generally, HS is bound by electropositive regions on a protein surface with significant electrostatic potential (Xu and Esko, 2014). To map the Hh electropositive regions *in vitro*, we characterized binding of the vertebrate Hh ortholog Shh to polyanionic heparin and HS coupled to FPLC affinity columns. We then analyzed Shh variants with mutated basic candidate amino acids using the same column-coupled matrices (Suppelemtary Table S1). Previous studies identified two electropositive Shh regions that strongly interact with HS, the CW-motif including highly conserved amino acids K33, R35, and K39 (Cardin and Weintraub, 1989; Farshi et al., 2011) and a second site consisting of amino acids K88, R124, R154, R156 and K179 (Whalen et al., 2013) [Figure 2A, shown is PDB: 3 m1n (Pepinsky et al., 2000)]. We added amino acid K46 to our screen because it is in close vicinity to the second HS binding site, and we also included basic amino acids R102 and K104 as a distant control site most likely not involved in HS binding. Key arginines (R) and lysines (K) were mutated to alanines (A) and proteins expressed in Bosc23 cells (a HEK293 derivative). The supernatant was then added to FPLC columns with coupled mouse embryo-derived HS (Kusche-Gullberg et al., 2012) (Figure 2B). As expected, Shh interacted strongly with the HS, and elution profiles of Shh^K88A^, Shh^K46A^, Shh^K46;88A^, and Shh^R102A;K104A^ were similar. Mutagenesis of the CW-motif abolished all protein binding to HS, and mutagenesis of K179 that is part of the second established HS binding site impaired HS/protein interaction as well. The latter finding was supported by reduced relative amounts of the HS-binding protein fraction if compared to the non-binders in the flow-through. Of note, while Shh variants differed in their binding kinetics, all proteins eluted at similar salt concentrations ranging from 1.1 to 1.5 M NaCl (Figure 2B). This indicated that, once formed, the strength of protein/HS interactions was not very much affected by the mutations. Moreover, we observed that all proteins (including Shh variants that lacked the CW-motif or with multiple HS-binding amino acids mutated into alanines) interacted more strongly with heparin. Because heparin is a HS variant with increased degrees of sulfation and negative charge (Figure 2C), our findings support that the bulk of free energy (ΔG) of the interaction is derived from the entropically favorable displacement of cations from HS upon Hh binding (Hileman et al., 1998; Xu and Esko, 2014), and not from modification-specific interactions (such as hydrogen bonding or van der Waals forces). Shh/HS interactions can therefore be imagined as closely resembling the unspecific interaction of Shh with a cation exchange resin. We note that this behavior is in stark contrast to previously observed shifts in the concentration of salt required for elution of other mutated extracellular proteins from heparin-Sepharose. Fibroblast growth factors FGF1, FGF2 and hepatic lipase, for example, bind very distinct modifications of HS by modification-specific interactions, and interferon-γ and interleukin-8 bind HS by domain-specific interactions (Thompson et al., 1994; Wong et al., 1995; Sendak et al., 2000). As a consequence, mutagenesis of basic amino acids in these proteins reduce the relative amounts of NaCl required to elute the proteins from the column. Therefore, similar HS binding strength of Shh and the Shh variants suggests that neither amino acid cluster is absolutely essential to maintain Shh interactions with HS, and that the interaction is not modification-specific.

### 2.3 Similar Hh variant signaling activities over short range *in vivo*

**FIGURE 3:**
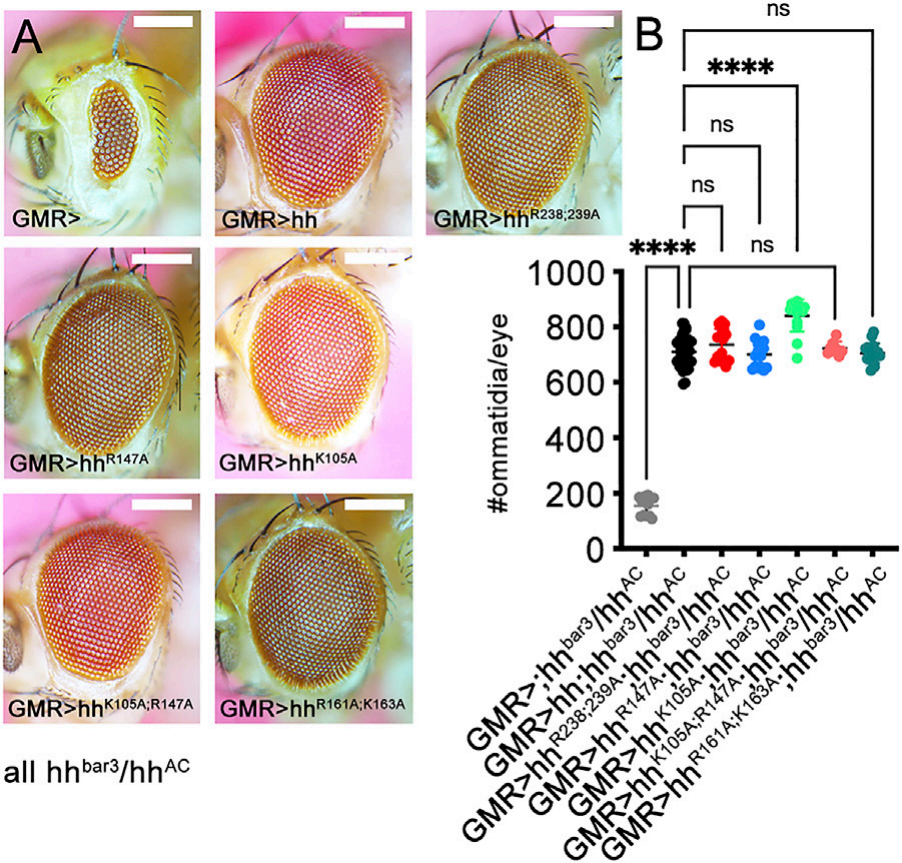
Hh short-range activity *in vivo* does not depend on established HS binding amino acids. Short-range Hh signaling in the eye disc determines the number of photoreceptors (ommatidia) in the adult *Drosophila* eye. **(A)** Discs lacking most Hh expression (hh^bar3^/hh^AC^) develop into small eyes (GMR>), but GMR-Gal4-controlled expression of hh and all tested hh variants restore eye development in this background. **(B)** Quantification of phenotypes. One-way ANOVA, two-tailed, Dunnett`s multiple comparisons *post hoc* test. ****: *p* < 0.0001, n.s, not significant (*p* > 0.046). No significant sex differences were observed. See Supplementary Table S2 for exact statistical results. Scale bars: 100 μm.

Following the confirmation of similar Shh and Shh variant expression, secretion to the cell surface, multimerization and activity in a cell-based bioassay (Supplementary Figures S2A–C), we generated transgenic flies by site-directed mutagenesis of *Drosophila* Hh. Hh and all Hh variants constructs were targeted into one specific attP-51C landing site (Bischof et al., 2007) to ensure similar gene expression, and their activities were compared in the developing *Drosophila* eye disc (Ma et al., 1993; Kastl et al., 2018). Development of the *Drosophila* eye depends on short-range Hh signaling that maintains and moves the morphogenetic furrow from posterior to anterior across the disc to pattern the tissue (Spratford and Kumar, 2014). Certain mutations within the hh gene, such as the viable hh^bar3^ allele and the loss-of function allele hh^AC^, impair morphogenetic furrow movement and thereby cause fewer ommatidial columns (Rogers et al., 2005; Kastl et al., 2018) (Figure 3A; Supplementary Table S2). We found that GMR-Gal4-controlled hh or hh^R238;239A^, hh^R147A^, hh^K105A;R147A^ and hh^R161A;K163A^ variant expression in the same genetic background (in GMR>hh; hh^bar3^/hh^AC^ flies) restored eye development to similar degree (Figures 3A, B; Supplementary Table S2). Of note, replacement of K105 for an alanine slightly increased protein biofunction in this system. Together, these findings confirmed similar production and secretion, oligomerization, release and binding of most transgenes to the Hh receptor Ptc *in vivo*. It also shows that site-directed mutagenesis of Hh amino acids corresponding to Ptc-binding K46 and K88 (Gong et al., 2018) (representing amino acids K105 and R147 in the *Drosophila* ortholog) or adjacent residues R238 and R239 do not affect Hh binding to Ptc, as this would have resulted in smaller eyes.

### 2.4 Impaired HS interactions variably affect Hh function at more distant target sites *in vivo*

**FIGURE 4:**
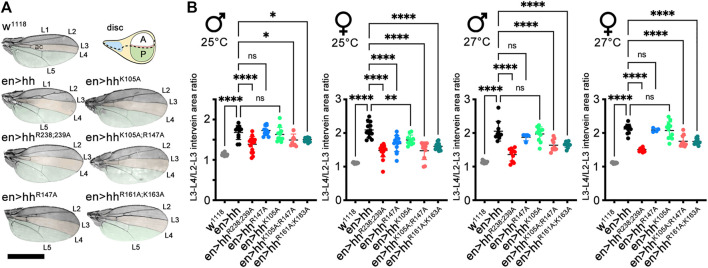
Hh and Hh variants expressed in the posterior wing disc compartment induce variable patterning of anterior wing structures. **(A)** Adult wings and schematic of a *Drosophila* third-instar wing disc. The posterior en-expression domain in the disc, and adult wing tissue arising from the posterior compartment are labeled in green. The anterior (A)/posterior (P) compartment border in the schematic is shown as a dashed line. Adult wings are shown with anterior up and proximal left. Longitudinal veins L1-L5 and the anterior cross vein (ac) are marked. Hh overexpression under en-control (en>hh) expands the anterior L3-L4 intervein region (orange) and apposes veins L2 and L3. **(B)** To quantify Hh activity at two different temperatures during development, the most proximal L3-L4 areas highlighted in orange were determined and the values divided by the L2-L3 areas. One-way ANOVA, two-tailed, Dunnett's multiple comparisons *post hoc* test. ****p ≤ 0.0001, ***p ≤ 0.001, **p ≤ 0.005, *p ≤ 0.01. Also see Supplementary Table S2 for exact statistical results. Scale bar: 1 mm.

The *Drosophila* wing disc is another well-established model system to analyze HS-regulated Hh biofunction. The wing disc differs from the eye disc in that Hh is expressed in the entire posterior wing disc compartment in response to the Engrailed (En) transcription factor [summarized in (Crozatier et al., 2004; Hartl and Scott, 2014)] and then moves anteriorly to reach ∼12 cell rows in the anterior wing disc compartment (Figure 4A, schematic, posterior is green, the anterior red stripe at the anterior/posterior border indicates Ptc-expressing cells that receive high Hh signals). In adult *Drosophila* wings, the position of the longitudinal L3-L4 veins results from the anterior Hh movement and signaling in the wing disc primordium, and thus provides one reliable read-out of the positional information provided by high Hh concentrations close to the source (Figure 4A, top left, the L3-L4 intervein area is labeled in orange, the wing area derived from the Hh-producing posterior wing disc compartment is labeled green) (Crozatier et al., 2004). Because development of the anterior L3-L4 intervein area depends on sufficient Hh spread over several cell diameters, en-Gal4 controlled overexpression of transgenic Hh in the posterior wing disc compartment expands the L3-L4 intervein space and, as a concomitant effect, reduces the L2-L3 intervein space [the L2-L3 area is determined by the Hh low-threshold target gene dpp (Figure 4A)] (Mullor et al., 1997; Strigini and Cohen, 1997; Lee et al., 2001; Crozatier et al., 2004). At 25°C, we found that the ratio between L3-L4 and L2-L3 intervein areas increased from 1.13 ± 0.03 and 1.1 ± 0.02 (male and female w^1118^) to 1.68 ± 0.03 and 2.1 ± 0.22 (en>hh, *p* < 0.001 for both sexes). This again confirmed that transgenic Hh was functional (Figure 4B), as observed before in the eye disc (Figure 3). In contrast to en>hh, overexpression of hh^R238;R239A^ under the same en-Gal4 control did not much change L3-L4 and L2-L3 intervein space ratios (1.39 ± 0.19 and 1.48 ± 0.18 for male and female en>hh^R238;239A^ flies, Figure 4B). This was because the size of the more anterior L2-L3 intervein field in en>hh^R238;239A^ (or under hh-Gal4 control, Supplementary Figure S3) also expanded, in notable contrast to the constriction of the same field in en>hh wings (Figure 4A). Expression of all other Hh variants under the same en>Gal4 control resulted in wing patterning phenotypes resembling those caused by hh overexpression. This suggests that, similar to what we have observed in the eye disc, Hh function in the wing disc was not much affected by most mutations, with the exception of amino acids R238 and R239 (Figures 4A, B, these amino acids correspond to Shh amino acid K179, Supplementary Table S1). This was also consistently observed in both sexes if flies were kept at 27°C to increase transgene transcription from the temperature-sensitive en-Gal4 promotor. We conclude from these findings that site directed mutagenesis of Hh amino acids R238 and R239 specifically diminished Hh high threshold patterning activity close to the A/P border in the wing disc, and increased intervein tissue formation in more anterior L2-L3 intervein positions (Supplementary Figure S3). Based on this unusual behavior – which indicates altered hh^R238;239A^ gradient range and slope – we focused on comparing en>hh function and en>hh^R238;239A^ function in all our subsequent studies.

### 2.5 Impaired direct HS interactions affect Hh target gene induction in the wing disc

**FIGURE 5:**
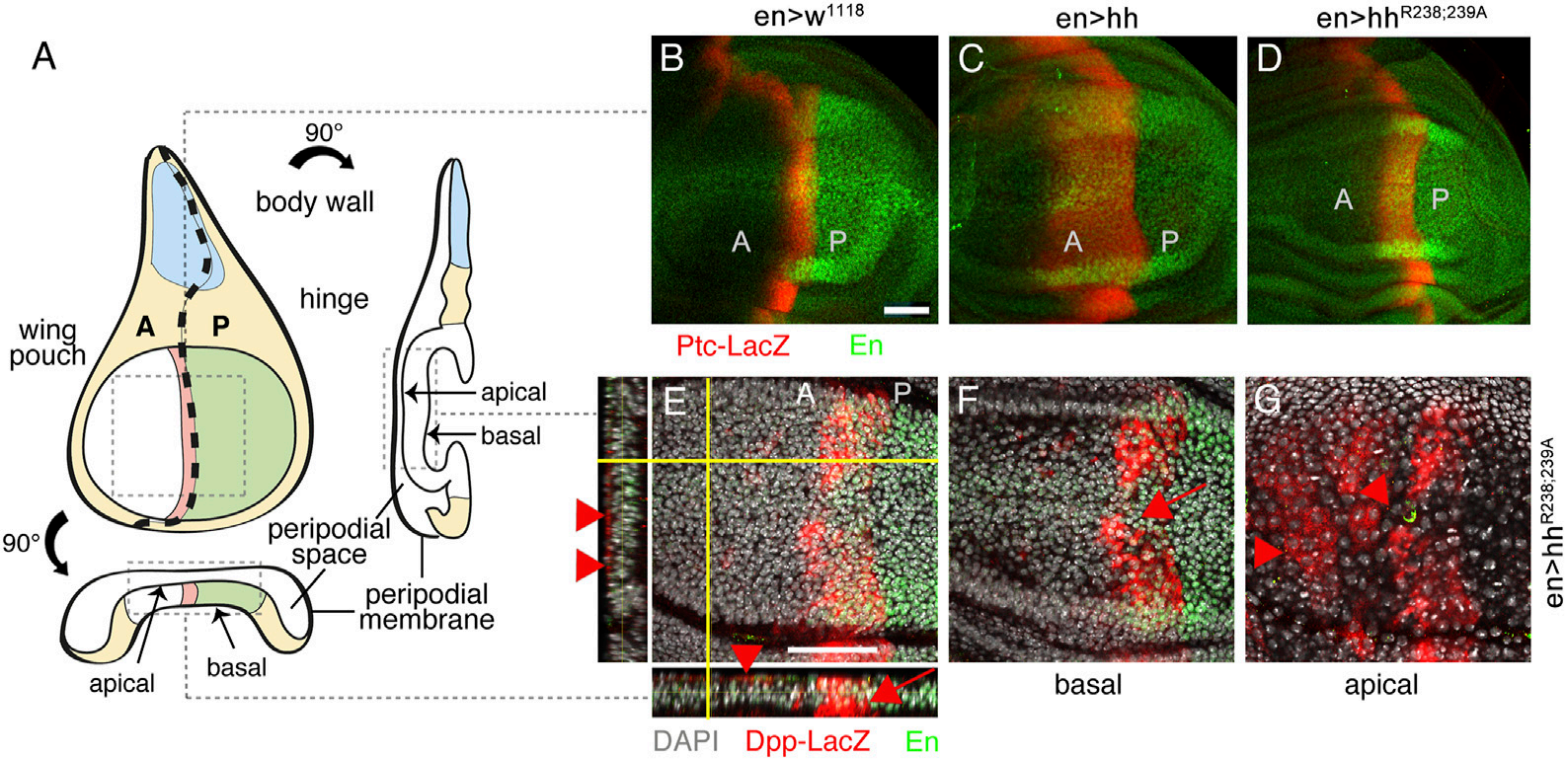
Different Hh variant activities in the developing wing disc. **(A)** An imaginal wing disc consists of an epithelial sheet of cells that form a sac-like infolding of the epithelium. Hh is produced in the posterior compartment of the wing disc under en control (labeled green) and moves across the anterior/posterior (A/P) boundary into the anterior compartment to induce Ptc and Dpp expression at the A/P border (labeled red). Frontal, orthogonal and axial wing disc views are shown. **(B–D)** Ptc-LacZ target gene expression (labeled red) in en>w^1118^, en>hh and en>hh^R238;239A^ wing discs. Note reduced relative Ptc-LacZ expansion in en>hh^R238;239A^ wing discs if compared with discs that overexpress Hh. **(E)** Views of a merged Z-stack of a disc expressing Hh^R238;239A^. In addition to expected Dpp-LacZ expression in the disc proper (arrow), Dpp-LacZ was also expressed at ectopic anterior sites of the peripodial membrane (arrowheads). **(F, G)** Confocal images of basal **(F)** and apical **(G)** layers taken from the same Hh^R238;239A^ expressing wing disc confirm ectopic Dpp-LacZ expression at the peripodial membrane. Ectopic Dpp-LacZ expression in this tissue was never observed in Hh overexpressing discs (Supplementary Figure S5). Anti-β-Gal staining visualized Ptc-LacZ and Dpp-LacZ (red). Anti-en-antibodies indicate the Hh-producing (posterior) compartment (labeled green). Scale bars: 50 μm.

As described before, Hh is expressed in the posterior wing disc compartment in response to the Engrailed transcription factor and spreads over significant distances into the anterior wing disc compartment (Schematic in Figure 5A). Here, rows of distant cells receiving minimal Hh signaling activate dpp transcription and cells receiving higher Hh signaling closer to the source–representing the cells that later give rise to the L3-L4 intervein field–activate the expression of ptc in addition to that of dpp (red stripe in Figure 5A). To determine the impact of Hh and Hh^R238;239A^ on the expression of both target genes, we crossed en>hh and en>hh^R238;239A^ with flies expressing Ptc-LacZ and Dpp-LacZ reporters (Basler and Struhl, 1994). As shown in Figure 5B, wild-type control flies express Ptc-LacZ in a normal stripe of cells just anterior to the A/P compartment boundary in response to endogenous Hh (red stripe), and the expression of additional transgenic Hh under en-control expands this stripe, as expected (Figure 5C). Consistent with the relatively smaller L3-L4 intervein area formed in en>hh^R238;239A^ wings, the stripe of Ptc-LacZ expression in the Hh^R238;239A^ disc is also smaller (Figure 5D): Compared with wild-type Hh, the width of Ptc-LacZ expression was reduced from 92 ± 10 μm (Hh) to 36 ± 2 μm (Hh^R238;239A^) (a 60% reduction, Supplementary Figure S4). Of note, we also found that wing discs from en>hh^R238;239A^ larvae express Dpp-LacZ ectopically in the peripodial membrane that overlays the wing disc proper (Figures 5E–G; Supplementary Figure S5), consistent with previously reported peripodial Dpp expression in response to overexpression of the Hh-activated transcription factor Cubitus interruptus (Pallavi and Shashidhara, 2005). Therefore, if compared to their similar activities in the developing eye disc, reduced Hh^R238;239A^ biofunction at the surface of the epithelial plane (Figure 5D) suggests that a fraction of Hh^R238;239A^ may have detached from the apical epithelial matrix (Figure 1, Supplementary Figure S1) and diffused away into the fluid-filled peripodial space (Figure 5A). This in turn may have induced ectopic Dpp expression at the apposed peripodial membrane (Pallavi and Shashidhara, 2005) (Supplementary Movie S1). We never observed ectopic Dpp-LacZ expression in wing discs overexpressing Hh under the same en-Gal4 control (*n* = 8), ruling out “leaky” transgene expression as one potential alternative reason (Supplementary Figure S5). Our findings therefore suggest that HS-binding Hh amino acids R238/239 (corresponding to Shh amino acid K179) constrict the morphogen to the apical epithelial surface and prevent Hh loss from the matrix into the fluid-filled peripodial space.

### 2.6 K179 substitution increases morphogen dissociation from a heparin-functionalized surface

**FIGURE 6:**
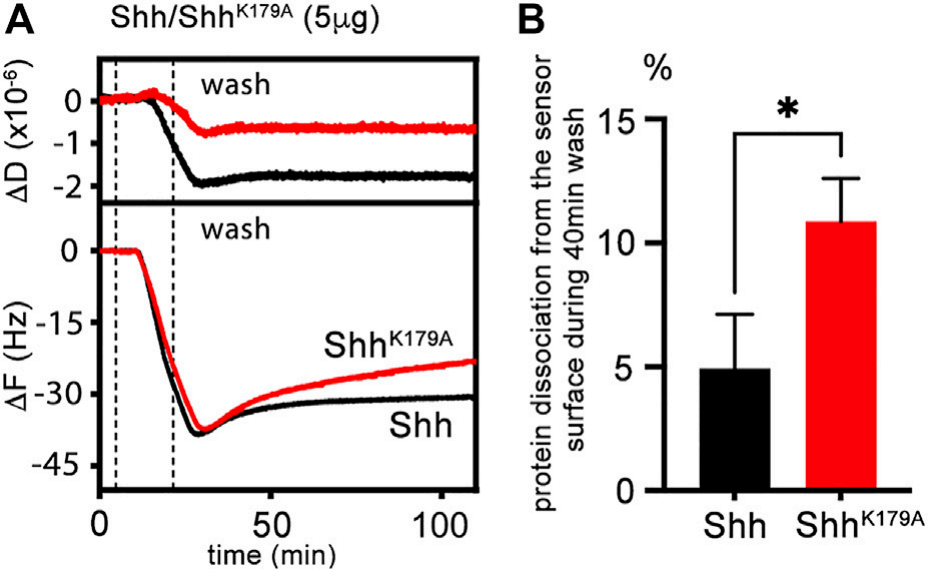
QCM-D measurements of untagged Shh interactions with heparin. **(A)** Schematic representation and real-time analysis of Shh binding to the functionalized QCM-D sensor surface assembled on a silica surface. The supported lipid bilayer exposes heparin to represent the HS model matrix. Shh binding decreases ΔF during the protein incubation step and decreases ΔD correspondingly, demonstrating protein binding and decreased softness of the functionalized matrix. Similar Shh^K179A^ loading of the functionalized matrix (-ΔF about −40 Hz in both cases) is associated with a less negative ΔD. The most parsimonious explanation for matrix softening is that Shh cross-links its components and that K179 of the second HS binding site participates in the process. Removal of this amino acid in Shh^K179A^ results in increased protein loss from the QCM-D sensor surface into the wash buffer (red line). **(B)** Graphical representation of Shh and Shh^K179A^ elution. Unpaired *t*-test, results from three (Shh^K179A^) and four (Shh) independent experiments are shown. *: *p* = 0.011. See Supplementary Table S2 for exact statistical results.

We tested the possibility of increased Hh^R238;239A^ loss from HS by quartz crystal microbalance with dissipation monitoring (QCM-D) as an *in vitro* method. QCM-D allows probing dynamic interactions of unlabeled macromolecules with surfaces in real time and with nanoscale resolution *in vitro* (the QCM-D principle is outlined in Supplementary Figure S6). We functionalized protein interaction surfaces using supported lipid bilayers containing 5% biotinylated lipids as a substitute of the plasma membrane, and streptavidin-linked highly sulfated biotinylated heparin, which served as a proxy for cell-surface HS. Like cell-surface HS attached to GPI-linked glypicans, streptavidin-linked heparin can rotate freely and move laterally on the sensor surface. We then added the protein ligand and monitored nanoscale mass changes upon protein adsorption to the heparin surface, as indicated by decreased resonance frequencies (-ΔF) of the sensor, and dissipation changes (ΔD) that provide information on viscoelastic layer properties, such as softening or stiffening of the layer (Supplementary Figure S6). Generally, increasing D indicates a more viscoelastic (softer) layer, while decreasing D indicates that the adsorbed layer becomes stiffer. We analyzed Shh and Shh^K179A^ instead of *Drosophila* Hh and Hh^R238;239A^ because the invertebrate proteins proved very difficult to produce and purify in the required amounts. As shown in Figure 6A, incubation of the QCM-D chip with Shh (black lines) induced a frequency shift (-ΔF) of about −40 Hz, indicating fast binding kinetics. This parameter reversed only slowly after washing the surface of the QCM-D chip with buffer, even after extended time periods (100 min, Figure 6A), showing that most Shh remained bound (5% ± 2.2% protein loss from the surface in the time between +40 min and +70 min after wash buffer injection, *n* = 4, Figures 6A, B). We also observed that the D of the layer decreased proportionally during Shh binding, suggesting a stiffened—cross-linked (Migliorini et al., 2015)—layer, consistent with the presence of two Shh binding sites for HS/heparin (Figure 6A) and providing one explanation for the low Shh k_off_ during the buffer wash. Next, we tested the capacity of unlabeled Shh^K179A^ variant (red line) to bind to the heparin-functionalized QCM-D chip surface (Figures 6A, B). Although we observed only slightly slower binding kinetics, heparin cross-linking by Shh^K179A^ was strongly reduced, as indicated by a smaller reduction of D during protein binding. We also observed an increase in protein k_off_ when washing the surface with buffer (Shh^K179A^: 11% ± 1.7% protein loss from the surface in the time between +40 min and +70 min after wash buffer injection, *n* = 3). This suggests that K179 does not contribute much to the initiation of heparin binding, but that this residue is important in polyvalent binding and stiffening of the heparin layer, as demonstrated by a smaller ΔD if compared to that caused by Shh (Figure 6A). We conclude from these findings that K179 is part of a polybasic site that interacts with a second heparin chain at the chip surface to reduce the k_off_ of the protein. In the wing disc, a similar function of Hh amino acids R238 and R239 may explain Hh^R238;239A^ loss from the matrix, diminished signaling at the A/P border, and Dpp expression in the peripodial membrane caused by the freely diffusing protein.

### 2.7 Impaired HS interactions affect Hh patterning robustness in the wing disc *in vivo*

**FIGURE 7:**
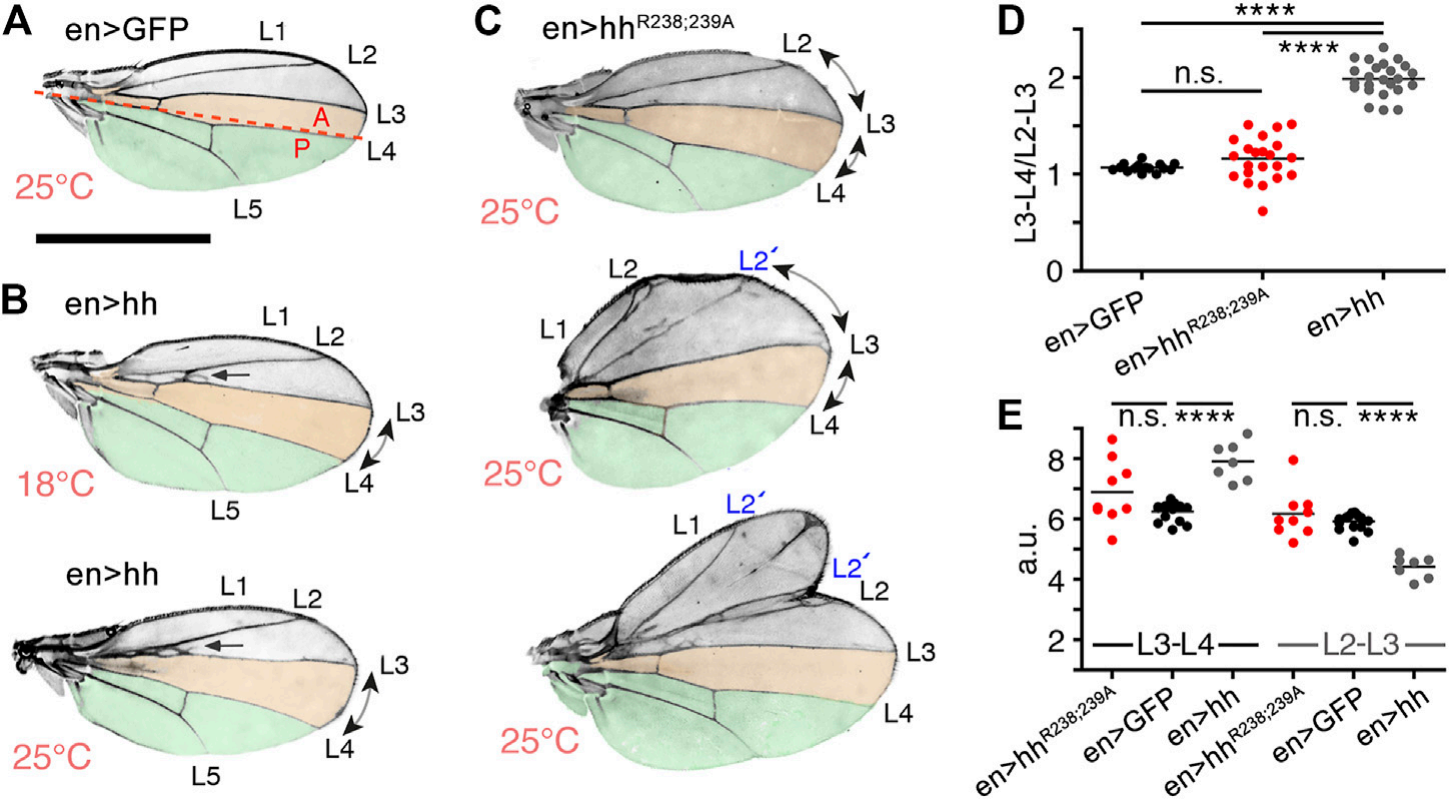
Substitution of HS-binding Hh amino acids affects *Drosophila* wing patterning. **(A)** En-GFP control flies only produce endogenous Hh in the posterior (P) wing disc compartment to directly pattern the later central domain of the adult wing (orange). The A/P boundary is indicated by the red dashed line. **(B)** Wing development in flies overexpressing Hh under en control at 18°C (B, weak Hh transgene expression) and 25°C (bottom, strong expression). The L3-L4 intervein region is always expanded in en>hh wings and the L2-L3 intervein region is always proximally apposed (indicated by arrows). **(C)** In en>hh^R238;239A^ wings at 25°C, both intervein regions expand, and the formation of additional longitudinal L2 veins in the anterior compartment of en>hh^R238;239A^ wings (labeled L2’) and mirror-image duplications of the anterior compartment (bottom wing) both support ectopic signaling as the underlying cause. Scale bar: 1 mm. **(D)** To quantify Hh activity, the most proximal L3-L4 areas highlighted in orange were determined and the values divided by the L2-L3 areas. One-way ANOVA, two-tailed, Turkey`s multiple comparisons *post hoc* test. ****p ≤ 0.0001, n.s, *p* > 0.01. **(E)**. L3-L4 areas and L2-L3 areas in en>GFP, en>hh and en>hh^R238;239A^ wings. One-way ANOVA, two-tailed, Turkey`s multiple comparisons *post hoc* test. ****p ≤ 0.0001, n. s, *p* > 0.01. See Table S2 for exact statistical results.

Stiffening of the heparin matrix, as observed in QCM-D, indicates that Shh simultaneously binds to more than one HS chain, and that dual HS interactions serve to tightly associate Hh with the epithelial plane. Contrary to what one would expect, we recently showed that such dual HS interactions and tight binding still allow for dynamic Hh movement in the gradient field to form functional gradients in *Drosophila* wing discs and eye discs *in vivo* (Gude et al., in press). This is because simultaneous interactions with two HS chains serve as a prerequisite for direct Hh transfer from one HS chain to the next. This avoids repeated steps of protein dissociation from HS, intermittent free diffusion, and re-association with HS that applies to proteins with only one HS binding site [called “restricted diffusion” (Stapornwongkul and Vincent, 2021)]. Impaired Hh^R238;239A^ interactions with HS may therefore have converted direct and uninterrupted HS-guided protein transport at the epithelial plane (Gude et al., in press) into an alternative on/off binding mode that may have led to significant morphogen loss into the overlaying, fluid-filled closed entity–the peripodial space (Figure 5A). Strong protein overexpression, as achieved by the Gal4/UAS system in this study, may therefore have enriched freely diffusing Hh^R238;239A^ in this entity high enough to induce target gene expression at ectopic sites that do normally not receive Hh. We support this possibility by striking wing mispatterning phenotypes in anterior parts of adult wings as a consequence of en-controlled hh^R238;R239A^ overexpression (Figures 7A–C). As shown before (Figure 4), the ratio between L3-L4 and L2-L3 intervein areas increased from 1.07 ± 0.04 (female en>Gal4, no Hh protein overexpression, at 25 °C) to 1.98 ± 0.17 (en>hh) (Figure 7D, Supplementary Table S2). In contrast, the L3-L4 and L2-L3 intervein area ratios in en>hh^R238;239A^ wings were only moderately changed (1.16 ± 0.23) (Figure 7D, Supplementary Table S2). Again, this is because L3-L4 intervein areas in en>hh wings were increased over those in en>hh^R238;239A^ wings, and L2-L3 intervein areas were slightly increased in en>hh^R238;239A^ wings but decreased in en>hh wings (Figures 4, 7E). In addition, in the most extreme cases, we also observed variably sized mirror-image duplications of the entire anterior compartment lacking the central L3-L4 domain (Figure 7C, bottom). This latter phenotype is similar to phenotypes reported in (Zecca et al., 1995; Jiang and Struhl, 1998) and indicates increased indirect patterning induced by aberrant expression of the low-threshold Hh target gene dpp. Indeed, Dpp misexpression is known to result in overgrowth of the anterior of the L2-L3 intervein compartment, duplications of veins 2 or 5, and mirror-image duplications (Capdevila and Guerrero, 1994). The link between our observed wing mispatterning phenotypes as a result of En-controlled hh^R238;R239A^ overexpression and aberrant Dpp signaling are in line with observed ectopic Dpp-LacZ expression in the peripodial membrane (Figures 5E–G). Note that anterior wing overgrowth is not likely a consequence of generally increased Hh^R238;R239A^ production, release or stability, as this would have also increased signaling in the eye disc (Figure 3). Alternative scenarios, e.g., “leaky” expression of the Hh transgene in anterior compartment cells of the wing disc can also be ruled out, as this would have led to mirror-image duplications of the entire anterior compartment, including the inter-L3-L4 field (Capdevila and Guerrero, 1994; Zecca et al., 1995; Tabata and Takei, 2004). The possibility of “leaky” en-Gal4-driven ectopic Hh^R238;R239A^ expression elsewhere also seems unlikely because Hh expression using the same driver line never caused anterior overgrowth and duplications, but consistently reduced L2-L3 intervein growth. Finally, the possibility of expanded Hh^R238;R239A^ signaling range caused by reduced Ptc receptor binding and protein internalization is ruled out by the observation that residue K179 is not required for Ptc binding (Gong et al., 2018), and the observation that Hh^R238;R239A^ bioactivity in the eye disc remained unimpaired. We conclude from our findings that selectively impaired HS binding of mutated proteins strongly affects Hh gradient range and robustness. This in turn implies that Hh gradient formation in the *Drosophila* wing disc requires direct Hh interactions with apically expressed HS.

## 3 Discussion

Morphogens organize cell fate decisions in an amazingly reproducible manner. To achieve this task, extracellular morphogen movement needs to overcome several constraints imposed by the need for gradient precision, scalability and robustness (Lander et al., 2002; Lander, 2013; Muller et al., 2013; Stapornwongkul and Vincent, 2021). Although it has been pointed out before that diffusion is one conceivable way to meet these demands (Lander et al., 2002; Lander, 2013), a major challenge is to envision how diffusing molecules can be confined to the planar surface of the *Drosophila* imaginal wing discs to limit leakage out of the tissue and to ensure that all molecules in the gradient field find their receptors (Kornberg and Guha, 2007).

One previously suggested solution to this problem is the encounter of morphogens with non-signaling binding partners at the epithelial surface, such as the HSPGs, to modulate diffusion-based morphogen gradients. However, this does not change the challenge to explain how the affinity for extracellular non-signaling binders can confine the morphogen to the planar surface and at the same time improve gradient dynamics and shape in the diffusion-based transport system [as discussed in (Stapornwongkul and Vincent, 2021)]. This is because reduced morphogen loss and extended gradient ranges are conflicting constrains: increasing the morphogen interactions with extracellular non-signaling binders (e.g., HSPGs) will hinder morphogen diffusion, and increasing the dissociation rate k_off_ of the interaction to facilitate transport will enhance morphogen leakage off the epithelial plane.

**FIGURE 8:**
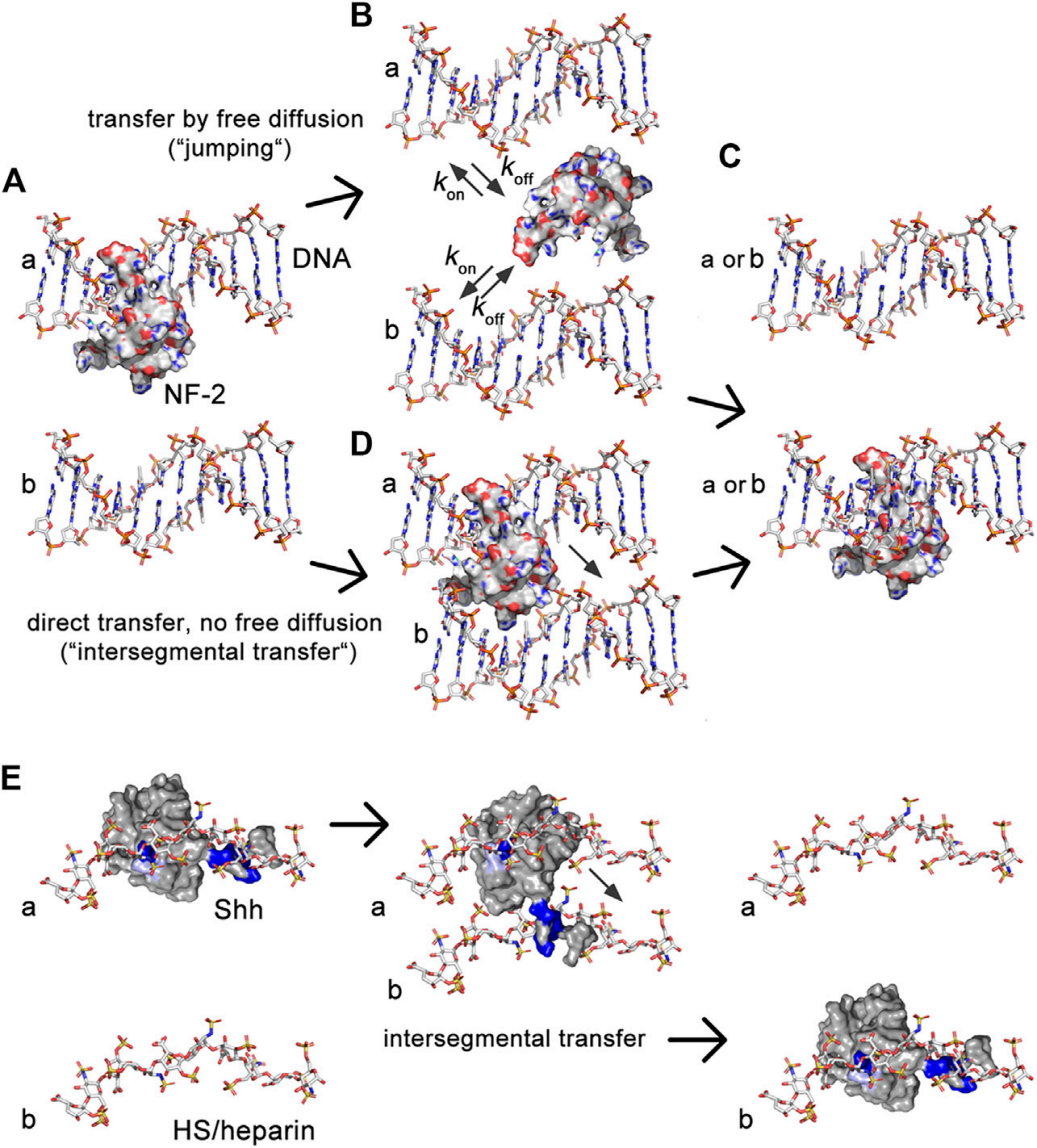
Illustration of the similarities between transport of intracellular DNA-binding proteins and extracellular HS-binding proteins. **(A)** DNA-binding proteins interact electrostatically with sugar-phosphate chains and exchange between them using two different mechanisms, called “jumping” and “intersegmental transfer.” Shown is the molecular structure of nuclear factor 2. **(B)** In the jumping mechanism, the protein fully dissociates from the first DNA strand **(A)** before binding to the second DNA strand [b in **(C)**] or before re-binding to the first **(A)**. **(D)** In the intersegmental transfer mechanism, two DNA-binding domains interact simultaneously with two DNA strands as a prerequisite to directly switch between them without a diffusible intermediary. **(E)** We recently showed that simultaneous Hh/Shh interactions with two extracellular sugar sulfate chains, as suggested by QCM-D, also serves as a prerequisite for intersegmental transfer (Gude et al., in press). Intersegmental transfer avoids intermittent free diffusion and subsequent Hh/Shh loss into the overlaying fluid-filled peripodial space. As a consequence, interference with HS-binding of the second site converts intersegmental transfer more into a jumping mode. Note that the first interaction site of DNA binding proteins often locates to a flexible tail, and that tailed DNA binders often switch through an intermediate in which the second binding site remains absorbed to one DNA while the flexible tail searches for the DNA acceptor (Vuzman et al., 2010). This “monkey bar” mechanism avoids intermittent steps of free protein diffusion and protein loss from DNA. We note that the first HS binding site of Hh/Shh (the Cardin-Weintraub motif) also locates to an N-terminal extended protein tail. This suggests that a similar search-and-switch mechanism may be used by the Hhs, and that aborted switching of our mutated protein may have converted it into a freely diffusible form. Shown are pdb structures 3iri (HS), 1bna (DNA), 1nk3 (nuclear factor 2) and 3 m1n (Shh). Structures are not to scale.

In the past, it has been shown that similar conflicting constraints have been elegantly solved by many intracellular proteins. Since the 1970s, it has been recognized that the relatively short time that DNA polymerases, transcription factors, nucleases and other DNA-binding proteins need to find their target cannot be explained by 3-D diffusion and random binding: The association with a specific DNA target sequence is about two orders of magnitude higher (Riggs et al., 1970) [summarized in (Barbi and Paillusson, 2013)]. This increase is achieved by the constriction of protein diffusion by non-specific, electrostatic interactions with the DNA backbone (Blainey et al., 2006; Elf et al., 2007) (Figure 8A). Such electrostatic constrictions prevent protein diffusion away from the DNA, yet do not keep the protein from moving along the axis of the double helix to find the specific DNA target sequence faster. This process is called sliding. Although DNA-binding proteins are able to switch between two DNA chains by free diffusion (Figures 8B, C), an additional important property of these proteins is their ability to simultaneously bind two DNA strands *via* two independent DNA binding sites (Barbi and Paillusson, 2013). This allows DNA-binders to directly switch from one strand to the next, a property called “intersegmental protein transfer” (Figure 8D). The advantage of intersegmental protein transfer is that it avoids the effectivity problems associated with 3-D diffusion and random binding that would apply to proteins with only a single interaction site–in particular the problem of protein loss from the DNA.

Analogous to this mechanism, we suggest that two HS binding sites constrict Hh to the extracellular matrix *via* unspecific electrostatic interactions (Figure 8E). The role of the second HS-binding site is to bind an adjacent HS chain to minimize k_off_ of the proteins, which may be particularly important during repeated direct Hh switching from one HS chain to the next during gradient formation (Gude et al., in press). Indeed, interfering with the full HS-binding capacity of the second HS binding site generates Hh^R238;239A^ that becomes subjected to “restricted diffusion” akin to on/off binders with one binding site, and that subsequently leak from the apical side of the epithelial plane (Ayers et al., 2010) into the liquid-filled peripodial space. This, in turn, strongly affects Hh gradient precision and robustness, because high threshold signaling close to the source was reduced and—in the case that Hh^R238;239A^ was strongly overexpressed by the Gal4-UAS system-ectopic signaling further away from the source was induced by the freely diffusing protein to varying degrees. In this regard, Hh^R238;239A^ resembles BMP4 mutant proteins that, if engineered to lack a stretch of basic amino acids that contribute to HS interactions, also have an increased signaling range (Ohkawara et al., 2002; Hu et al., 2004). QCM-D confirmed that the role of the second conserved Shh HS binding site is to cross-link heparin, at least temporarily during the process of switching to minimize the k_off_. This is in line with the known ability of other growth factors and chemokines to cross-link HS (Migliorini et al., 2015), raising the possibility that HS-defined “diffusible zones” as for extracellular Hh may also exist for other morphogens, growth factors, and chemokines (Migliorini et al., 2015; Dyer et al., 2017). HS-defined “diffusible zones” in developing tissues or during inflammation–not only on epithelial planes in two dimensions, but possibly also in compact tissues in three dimensions—are therefore likely to regulate temporal-spatial signaling by trapping molecules with the opposite charge while forcing molecules with the same charge into different zones.

We note that HSPG-defined “diffusible zones” can explain largely maintained wing morphology and landmarks upon En-controlled overexpression of transgenic Hh. This applies specifically to anterior wing structures that undergo surprisingly little morphological alterations in this system. Our interpretation is that tight HS constriction of Hh spread prevents oversaturation of the system, because diffusion is effectively limited to the A/P border and because source and sink of Hhs within this area are tightly controlled (Chen and Struhl, 1996; D'Angelo et al., 2015; Incardona et al., 2000; Marigo and Tabin, 1996; Torroja et al., 2004). We still observe moderate wing overgrowth that affects the posterior and even more so the anterior L1-L2 compartments (Figures 7A, B). This may indicate a direct Ptc-dependent translation of the Hh signaling amplitude into cell proliferation within the anterior compartment. Indeed, cell lineage analyses have previously shown that Ptc-expressing target cells at the A/P border of L3 larva wing discs are the youngest in birth order, yet can later be detected in more anterior regions of the wing disc (Evans et al., 2009). This observation supports that sustained proliferation of Ptc-expressing cells and the available Hh signaling amplitude at the A/P compartment boundary may be directly linked. The posterior compartment does not have such a boundary-focused proliferation pattern, and may therefore underly a different mode of growth control.

HSPG-defined “diffusible zones” can also explain anterior wing overgrowth upon en-Gal4-controlled Hh^R238;239A^ overexpression *via* the partial breakdown of the tight two-dimensionalization of signaling at the A/P border, caused by diminished HS binding of this variant. This enriches Hh^R238;239A^ in the fluid-filled peripodial space that, because it represents a closed entity, may allow for sufficient Hh signaling amplitudes at more anterior sites of the disc. This, in turn, may have resulted in the observed anterior wing overgrowth (Figure 7C). Wing duplications, as also shown in Figure 7C, may have originated from soluble Hh^R238;239A^ rebinding–which we note is not impaired (Figure 6A)—or “trapping” into HSPG-defined anterior diffusible zones, in turn forming ectopic signaling centers. A fold or groove in the 3D organization of the imaginal disc can also be perceived to form such a trap. Because squamous peripodial epithelia are largely polyploid, it is unlikely that they have directly contributed to anterior wing overgrowth or the observed duplications in diploid tissues, despite the observed ectopic dpp expression in this tissue. However, we cannot rule out that Dpp released from the peripodial epithelium may have indirectly contributed to the observed phenotypes.

## 4 Materials and methods

### 4.1 Cloning and expression of recombinant Shh and Hh

We used murine Shh (Dierker et al., 2009) and Hh sequences (nucleotides 1–1416, corresponding to amino acids 1–471 of *D. melanogaster* Hh) that were generated from cDNA by PCR using primer sequences that can be provided upon request. PCR products were inserted into pENTR for sequence confirmation and subsequently into pUAST for protein expression in S2 cells or the generation of transgenic flies. Mutations were introduced *via* the QuickChange Lightning site-directed mutagenesis kit (Stratagene, La Jolla, United States). S2 cells were cultured in Schneider’s medium (Invitrogen, Carlsbad, United States) supplemented with 10% fetal calf serum and 100 μg/mL penicillin/streptomycin. The cells were transfected with constructs encoding Hh and Hh variants together with a vector encoding an actin-Gal4 driver using Effectene (Qiagen, Hilden, Germany) and cultured for 36 h in Schneider’s medium before protein was harvested from the supernatant.

### 4.2 Analysis of transgenic *Drosophila* Fly lines

The following fly lines were used: En-Gal4e16E (En-Gal4): P(en2.4-GAL4)e16E, FlyBaseID FBrf0098595; Hh-Gal4: P(Gal4)hh^Gal4^ (FlyBase FBti0017278, Bloomington #67046); GMR17G12 (GMR17G12-Gal4): P(y[+t7.7]w[+mC] = GMR17G12-Gal4)attP2, Bloomington stock#45433 (discontinued); dpp-reporter: En-Gal4e16E/CyO; dpp-LacZ/Tm6B (dpp-LacZ (Exel), Bloomington stock #8404); ptc-reporter: yw; en-Gal4e16E/CyO^WeeP^;ptc-LacZ/Tm6B (ptc-LacZ reporter flies were kindly provided by Jianhang Jia, University of Kentucky, USA). Reporter lines were crossed with flies homozygous for UAS-Hh or UAS-Hh^R238;239A^ at 25°C and 27°C. Note that, at these temperatures, the Gal4-UAS system results in the expression of transgenes at much higher levels than endogenous tissue-specific promotors. Ectopic Hh expression in the morphogenetic furrow of the eye disc was conducted by crossing the following fly lines: UAS-Hh/CyO^WeeP^;Hh^AC^/Tm6B and GMR-Gal4/GMR-Gal4; Hh^bar^/Hh^bar^ (hypomorphic hh^bar3^: FlyBase ID FBal0031487). Resulting GMR > Hh^bar^/Hh^AC^ flies that additionally carry our transgenes under UAS-control were analyzed with a Nikon SMZ25 microscope. GMR; Hh^bar^/Hh^AC^ flies served as negative controls; OregonR or UAS-GFP expressing flies served as positive controls. Transgenic flies were generated by PhiC31 integrase-mediated transgenesis to the attP C51C site (BestGene, Chino Hills, United States). All Hh cDNAs cloned into pUAST-attP were first expressed in *Drosophila* S2 cells to confirm correct protein processing and secretion. Wings of the adult F1 were collected and mounted in Hoyers medium, and total intervein areas and total wing areas were quantified by MoticImage software. All fly crosses and maintenance were conducted at 25°C or 18°C, as indicated.

### 4.3 Protein purification and analysis by gel filtration

Proteins were resolved by reducing 15% SDS-PAGE and immunoblotted onto PVDF membranes. The immobilized Hh proteins were detected with a primary polyclonal anti-Hh antiserum (rabbit IgG, Santa Cruz Biotechnology, United States) and visualized with a secondary peroxidase-conjugated donkey anti-rabbit IgG (Dianova, Hamburg, Germany) followed by chemiluminescent detection. The signals were quantified with ImageJ software. Gel filtration analysis was carried out by FPLC (Äkta protein purifier, GE Healthcare, United States) on a Superdex200 10/300 GL column (Pharmacia) equilibrated with PBS at 4°C. Eluted fractions were TCA-precipitated, resolved by SDS-PAGE and immunoblotted. Signals were quantified using ImageJ and values expressed relative to the strongest signal, which was set to 100%.

### 4.4 Cloning of Shh variants and expression in *Escherichia coli*

After we cloned ShhN wt (Δ191-198) into pGEX4T1, QuickChange mutagenesis was used to substitute a thrombin cleavage site of the vector with a TEV protease cleavage cite (peptide sequence: ENLYFQS). This template was then used to generate Shh mutant variant sequences by QuickChange mutagenesis. *E. coli* BL21 were transformed by using heat shock transformation and plated out on ampicillin LB agar plates. Overnight cultures were grown in 50 mL TB medium in a shaking incubator at 37°C and 150 rpm. The next day, 400 mL TB medium in 1000 mL shake flasks was inoculated with 15 mL of the overnight culture and again incubated at 37°C and 150 rpm. After an OD_600_ of ∼1.2 was reached, expression of Shh or Shh variant proteins was induced by isopropyl-β-D-thiogalactopyranosid (IPTG) to a final concentration of 400 mM. Two hours later, the same amount of IPTG was added and the incubation continued for another 4 h. Cells were harvested in 50 mL falcon tubes and centrifuged at 4,800 rpm and 6°C for 20 min. The supernatant was discarded and the step repeated four more times. Cell pellets were stored at −20°C until further use.

### 4.5 Purification of *E. coli* produced ShhN for QCM-D

Reagents and solutions were kept on ice at all times. Cell pellets were carefully thawed on ice and cells resuspended in 7 mL PBS supplemented with 4 µL NP-40 (Thermo Scientific) and 100 µL cOmplete protease inhibitor cocktail (Sigma-Aldrich), 1 µL DNase I (10 mg/mL), lysozyme (Roth), and 800 µL glycerol (Roth). Cells were lysed by sonication (60% duty cycle, 70% amplitude) for 3 min × 1 min with cooling steps on ice for 1 min between each step. Lysed cells were centrifuged for 20 min at 4,800 rpm and at 6°C. The supernatant was first sterile filtered through a 0.45 µm filter and subsequently through a 0.2 µm filter. The filtered solution (5 mL) was then applied to an Äkta System (GE Healthcare) by using 1 mL GSTrap High Performance columns (Cytiva) and a flow rate of 1 mL/min. The columns were washed with PBS for 30 min (1 mL/min buffer flow). The fusion protein was eluted with 10 mM glutathione in PBS for 15 min at a flow rate of 1 mL per min and fractionated. Fractions 9 and 10 were pooled and incubated with 10 units ProTEV Plus (Promega) overnight at 30°C and 400 rpm to remove the Shh from the GST. The next day, Shh was dialyzed against cold MilliQ water by using a Slide-A-Lyser cassette (molecular weight cutoff 3.5 kDa, ThermoFischer Scientific). After dialysis, samples were taken for SDS-PAGE analysis to determine protein purity and concentration by using BSA standards of known concentration followed by densitometric analysis. The remaining samples were aliquoted, lyophilized, and stored at −80°C until further use.

### 4.6 Synthesis of biotinylated heparin for QCM-D analyses

Biotinylated heparin was synthesized by adapting a previously reported procedure (Thakar et al., 2014). First, a solution containing heparin (4 mM, Sigma-Aldrich), 10 mM acetate buffer (made from glacial acetic acid (Carl Roth, Karlsruhe, Germany) and sodium acetate (Sigma-Aldrich) at pH 4.5) and aniline (100 mM, Sigma-Aldrich) was prepared. Biotin-PEG_3_-oxyamine (3.4 mM, Conju-Probe, San Diego, United States) was added to the heparin solution and allowed to react for 48 h at 37°C. The final product was dialyzed against water for 48 h by using a dialysis membrane with a 3.5 kDa cutoff. The final solution was then lyophilized and stored at −20°C. For further use, the conjugates were diluted to the desired concentrations in buffer. The obtained biotinylated heparin was characterized by biotin-streptavidin binding assays using QCM-D. The average mass of the heparin isolate, when anchored to the surface, was estimated at 9 kDa (∼18 disaccharide units) by QCM-D analysis (Srimasorn et al., 2022).

### 4.7 Preparation of small unilamellar vesicles (SUVs)

SUVs were prepared by adapting reported procedures (Bartelt et al., 2018; Di Iorio et al., 2020). A mixture of lipids composed of 1 mg/mL 1,2-dioleoyl-sn-glycero3-phosphocholine (DOPC, Avanti Polar Lipids) and 5 mol% of 1,2-dioleoyl-sn-glycero-3-phosphoethanolamine-N-(cap biotinyl) (DOPE-biotin, Avanti Polar Lipids) was prepared in chloroform in a glass vial. Subsequently, the solvent was evaporated with a low nitrogen stream while simultaneously turning the vial in order to obtain a homogenous lipidic film. The residual solvent was removed for 1 h under vacuum. Subsequently, the dried film was rehydrated in ultrapure water to a final concentration of 1 mg/mL and vortexed to ensure the complete solubilization of the lipids. The lipids were sonicated for about 15min until the opaque solution turned clear. The obtained SUVs were stored in the refrigerator and used within 2 weeks.

### 4.8 QCM-D measurements

QCM-D measurements were performed with a QSense Analyser (Biolin Scientific, Gothenburg, Sweden) and SiO_2_-coated sensors (QSX303, Biolin Scientific). The measurements were performed at 22°C by using four parallel flow chambers and one peristaltic pump (Ismatec, Grevenbroich, Germany) with a flow rate of 75 µL per min. The normalized frequency shifts ΔF, and the dissipation shifts ΔD, were measured at six overtones (*i* = 3, 5, 7, 9, 11, 13). The fifth overtone (*i* = 5) was presented throughout; all other overtones gave qualitatively similar results. QCM-D sensors were first cleaned by immersion in a 2wt% sodium dodecyl sulfate solution for 30min and subsequently rinsed with ultrapure water. The sensors were then dried under a nitrogen stream and activated by 10 min treatment with a UV/ozone cleaner (Ossila, Sheffield, United Kingdom). For the formation of supported lipid bilayers (SLBs), after obtaining a stable baseline, freshly made SUVs were diluted to a concentration of 0.1 mg/mL in buffer solution (wash buffer A, 50 mM Tris, 100 mM NaCl (Sigma Aldrich) at pH 7.4) containing 10 mM of CaCl_2_ directly before use and flushed into the chambers. The quality of the SLBs was monitored *in situ* to ascertain high-quality SLBs were formed, corresponding to equilibrium values of ∆F = −24 ± 1 Hz and ∆D < 0.5 × 10^−6^. Afterward, a solution of streptavidin (Sav; 150 nM) was passed over the SLBs, followed by the addition of biotinylated heparin (10 μg per mL). Each sample solution was flushed over the QCM-D sensor until the signals equilibrated and subsequently rinsed with wash buffer A (see above). Before the addition of Shh protein (wild type and mutants) solutions, the flow rate was reduced to 20 µL per min.

### 4.9 Preparation of HS for FPLC analyses

1 g (wet weight) C57/Bl6 embryonic day 18 mouse embryos or *D. melanogaster* 1^st^–3^rd^ instar embryos were homogenized and digested overnight in 320 mM NaCl and 100 mM sodium acetate (pH 5.5) containing 1 mg/mL pronase at 40°C. The digested samples were diluted 1:3 in water and 2.5-mL aliquots were applied to DEAE Sephacel columns. HS was eluted and applied to PD-10 (Sephadex G25) columns (GE Healthcare), lyophilized, redissolved in 20 μL water, digested with chondroitinase ABC overnight as indicated, and again purified by DEAE chromatography. Samples were diluted and again applied to PD-10 columns prior to lyophilization. β-elimination of peptides was omitted from this purification protocol to allow for HS coupling to NHS-activated Sepharose *via* the attached peptides. We confirmed efficient HS coupling to NHS-activated Hi-Trap FPLC columns by using soluble alkaline phosphatase-coupled Fgf8 and VEGF as previously described (Farshi et al., 2011). HS binding of Shh/Hh was then determined by FPLC (Äkta protein purifier). Samples were applied to the columns in the absence of salt, and bound material was eluted with a linear 0–1 M NaCl gradient in 0.1 M phosphate buffer (pH 7.0). Eluted fractions were quantified as described above. Shh and Hh binding to heparin columns (GE Healthcare) was carried out with the same protocol, except for elution in a linear 0–1.5 M NaCl gradient in 0.1 M sodium phosphate buffer (pH 7.0).

### 4.10 Confocal microscopy

Transfected S2 cells were grown on cover slips in 12-well plates. At 48 h after transfection, cells were washed with PBS, fixed with 4% PFA in PBS at room temperature for 10 min, and blocked with 1% BSA in PBS for 30 min. Cells were incubated with primary polyclonal anti-HhN antiserum (rabbit IgG, Santa Cruz Biotechnology, United States) at 4°C for 12h, washed 3 times with PBS and incubated with secondary Cy3 donkey anti-rabbit IgG antibodies (1:300) (Dianova) for 2 h at room temperature. DAPI (1 μg/mL) was added to all incubations. Cells were washed 3 times with PBS and mounted in mounting medium (Vector Laboratories). Wing discs were fixed, permeabilized and stained with anti-β-galactosidase antibodies (Cappel, MP Biomedicals) and Cy3-conjugated goat-α-rabbit antibodies (Jackson Immuno Research). Posterior Hh-producing cells were detected with monoclonal antibodies directed against engrailed (4D9, DSHB) and Alexa488-conjugated donkey-α-mouse antibodies (Thermo Fisher). Images were taken on an LSM 700 Zeiss confocal microscope with ZEN software. Maximum intensity projections are shown and individual sections are shown where indicated. Orthogonal views were created using ImageJ software.

### 4.11 Protein bioactivity

Differentiation of C3H10T1/2 osteoblast precursor cells was used as a read-out to determine Hh bioactivity. Prior to analysis, protein aliquots were immunoblotted to confirm comparable protein amounts and protein integrity before use in the activity assay. Remaining conditioned media were then sterile filtered and applied to C3H10T1/2 (Nakamura et al., 1997) cells in 15-mm plates. Cells were lysed 5 days after induction (20 mM Hepes, 150 mM NaCl, 0.5% Triton X-100, pH 7.4) and the amount of alkaline phosphatase produced as a response of Hh-induced osteoblast differentiation was measured at 405 nm with 120 mM p-nitrophenolphosphate (PNPP, Sigma) in 0.1 M glycine buffer, pH 10.4. All assays were performed in triplicate.

### 4.12 Immunoelectron microscopy

Wing discs of Shh-expressing transgenic flies were fixed overnight at 4°C in 4% paraformaldehyde/glutaraldehyde, washed in PIPES, and dehydrated in a graded ethanol series (30% EtOH, 4°C, 45 min; 50% EtOH, −20°C, 1 h; 70% EtOH, −20°C, 1 h; 90% EtOH, −20°C, 1.5 h; 100% EtOH, −20°C, 1.5 h; 100% EtOH, −20°C, 1.5 h). Dehydrated cells were embedded in Lowicryl K4M embedding medium by using the Lowicryl® K4M Polar Kit (Polysciences). Cells were then embedded in gelatin capsules, centrifuged twice for 15 min at 1,500 rpm, and incubated overnight at −35°C. For polymerization, the resin was UV irradiated for 2 days at −35°C. The embedded samples were cut into 60-nm sections, washed in PBS containing 5% BSA (pH 7.4), and incubated for 2 h in PBS-BSA containing primary antibodies (α-Shh antibodies from R&D, GeneTex, and Cell Signaling at 1:20 dilution). Samples were washed five times in PBS-BSA and once in Tris-BSA. Secondary antibodies conjugated to 5-nm and 10-nm gold nanoparticles were diluted in Tris-BSA buffer and incubated with the cell sections for 1 h. Afterwards, samples were washed five times in Tris-BSA and once in dH_2_O. Contrasting was done with 2% uranyl acetate (15 min) and Reynold’s lead citrate (3 min). Finally, immunogold-labeled cell sections were analyzed by using a transmission electron microscope (CM10, Philips Electron Optics).

### 4.13 Bioanalytical and statistical analysis

Statistical analysis was performed in GraphPad Prism version 950. Applied statistical tests, post hoc tests and number of independently performed experiments are stated in the figure legends. A *p*-value of <0.05 was considered statistically significant. Error bars represent the s.d. of the mean.

## Data Availability

The original contributions presented in the study are included in the article/Supplementary Material, further inquiries can be directed to the corresponding author.
