# Supplementary material for: Drosophila hedgehog signaling range and robustness depend on direct and sustained heparan sulfate interactions
Source: Front Mol Biosci. 2023 Feb 22;10:1130064. doi: 10.3389/fmolb.2023.1130064 (PMC9992881; doi:10.3389/fmolb.2023.1130064)
Supplement: Supplementary file 1 [file DataSheet1.PDF]

Supplementary Material

Drosophila hedgehog signaling range and robustness depend on direct and sustained heparan sulfate interactions

Dominique Manikowski[1], Georg Steffes[2], Jurij Froese[1], Kristina Ehring[1], Fabian Gude[1], Daniele Di Iorio[1], Seraphine V. Wegner[1] & Kay Grobe[1*]

*** Correspondence:** Dr. Kay Grobe: kgrobe@uni-muenster.de

1 Supplementary Data

Supplemental Movie 1. 3-D rendered Z-stacks demonstrating ectopic Dpp-LacZ production (red) at the peripodial membrane overlaying the Hh-producing wing disc proper (the Hh producing compartment is labeled green).

2 Supplementary Figures and Tables

2.1 Supplementary Figures

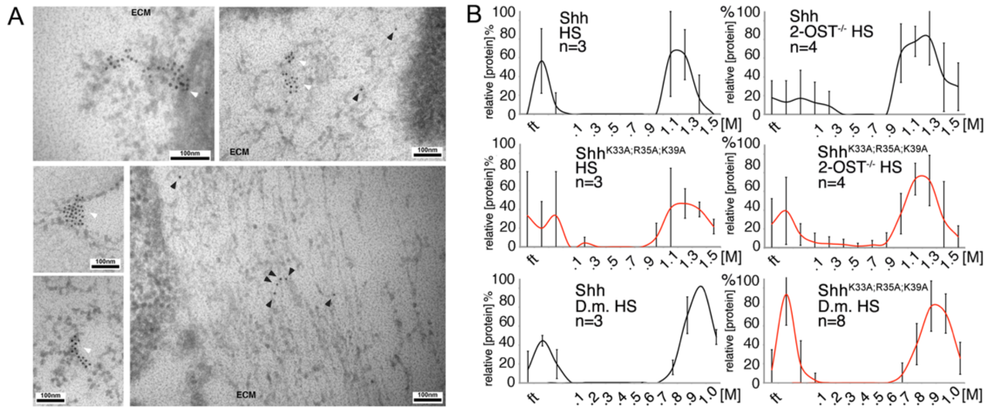

Figure supplement 1. Shh associates with the wing disc extracellular matrix. (A) Transmission electron micrograph of the apical columnar wing disc epithelium and overlying peripodial space. ECM: fibrillar extracellular matrix meshwork. Anti-Shh immunogold labeling detects untagged Shh closely associated with the fibrillar extracellular meshwork overlaying the epithelial surface (black arrowheads). **(B)** We tested Shh binding to physiological HS isolated from mouse embryos (HS), from mouse embryos deficient in 2-*O* sulfotransferase expression (2-OST$^{-/-}$ HS), and from fly larvae (D.m. HS) and then coupled to fast protein liquid chromatography (FPLC) columns. D.m. HS differs from heparin in its degree of sulfation: Both consist of single, extended *N*-sulfated domains, but D.m. HS has very low *O*-sulfate content. D.m. HS is also different from vertebrate HS that consists of highly *N*- and *O*-sulfated domains separated by unmodified domains (Kusche-Gullberg et al., 2012). HS affinity chromatography showed that Shh bound to all HS preparations and always eluted at 0.8 M-0.9 M NaCl,

suggesting that Hh interactions with HS are non-specific electrostatic interactions. A Shh variant lacking Cardin-Weintraub residues K33, R35 and K39 also bound to all forms, suggesting that the second HS binding site contributes strongly to the interactions. We conclude from these findings that TEM in (A) may show physiologically relevant interactions between overexpressed Shh and D.m. HS in the matrix.

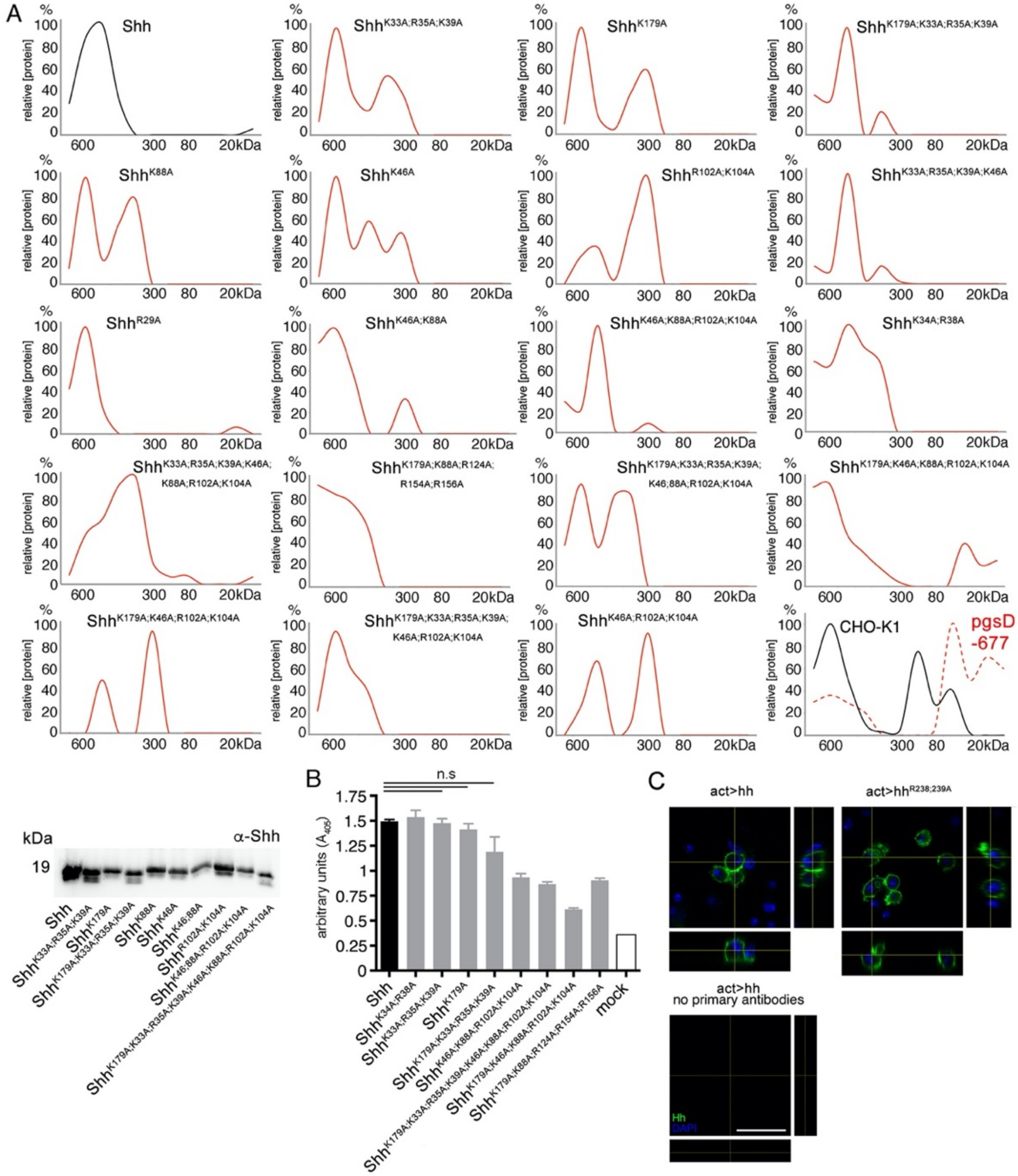

Figure supplement 2. Similar multimerization, signaling and secretion of Shh protein variants. (A) Gel filtration analysis of Shh and variant proteins confirmed unimpaired expression and HS-dependent multimerization of all proteins *in vitro*. HS-dependent multimerization was confirmed by Shh expression in chinese hamster ovary (CHO-K1) cells and pgsD-677 mutant cells that lack HS expression at the cell surface. Bottom: Shh and Shh variant proteins are expressed and secreted to similar levels. **(B)** Unimpaired signaling activity of Shh mutants. C3H10T1/2 osteoblast precursor cells

were incubated with equal amounts of wild-type and mutated Shh variants, and the relative amounts of Hh-induced alkaline phosphatase activity were determined as a readout for C3H10T1/2 differentiation and hence for biological activity of the morphogen variants. **(C)** Confocal analysis of *Drosophila* S2 cells expressing Hh and Hh$^{R238;239A}$. All proteins were expressed at comparable levels and locate at the cell surface (green). Cells were stained with α-Hh antibodies (1:250) and Cy3-labeled anti-rabbit IgG (1:600) under non-permeabilizing conditions, DAPI staining is shown in blue. Merged confocal stacks are shown. Representative results in orthogonal view are shown from experiments repeated at least twice. Scale bar: 20 μm.

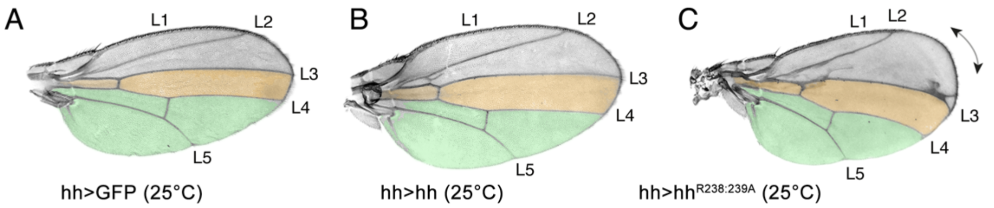

Figure supplement 3. Anterior wing mispatterning as a consequence of hh-Gal4-driven posterior Hh$^{R238;239A}$ overexpression. (A) A hh>GFP control wing is shown. Adult wings are shown with anterior up and proximal left. Longitudinal veins L1-L5 are marked. Tissues developing from the posterior Hh-producing wing disc compartment are labeled green (this area develops independently of the Hh signal). Central L3-L4 intervein tissue (orange) derives from Hh-receiving cells at the A/P border, and the L2-L3 intervein field is indirectly patterned by low-threshold Hh induction of Dpp. **(B)** Adult wing phenotype resulting from hh>Gal4 controlled Hh overexpression. **(C)** Adult wing phenotype resulting from hh>Gal4 controlled Hh$^{R238;239A}$ overexpression. The double arrow denotes expanded L2-L3 intervein tissue.

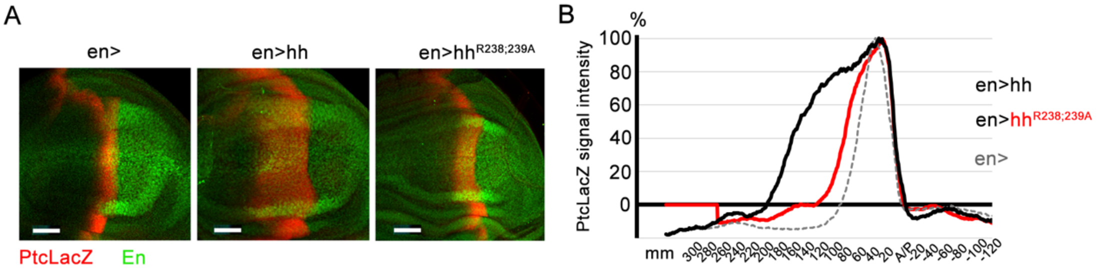

Figure supplement 4. En-Gal4-expressed Hh$^{R238;239A}$ fails to signal at the A/P border. (A) As shown in Fig. 5, Ptc-LacZ expression is normally restricted to a stripe in the anterior compartment adjacent to the A/P border. En-controlled expression of Hh expands the width of this stripe, indicating an expanded high-threshold activity range of Hh. Hh$^{R238;239A}$ expands the stripe of high morphogen activity much less. Scale bars: 50 μm. **(B)** Quantification of posterior-anterior Ptc-LacZ width, as shown in (A) and Fig. 5.

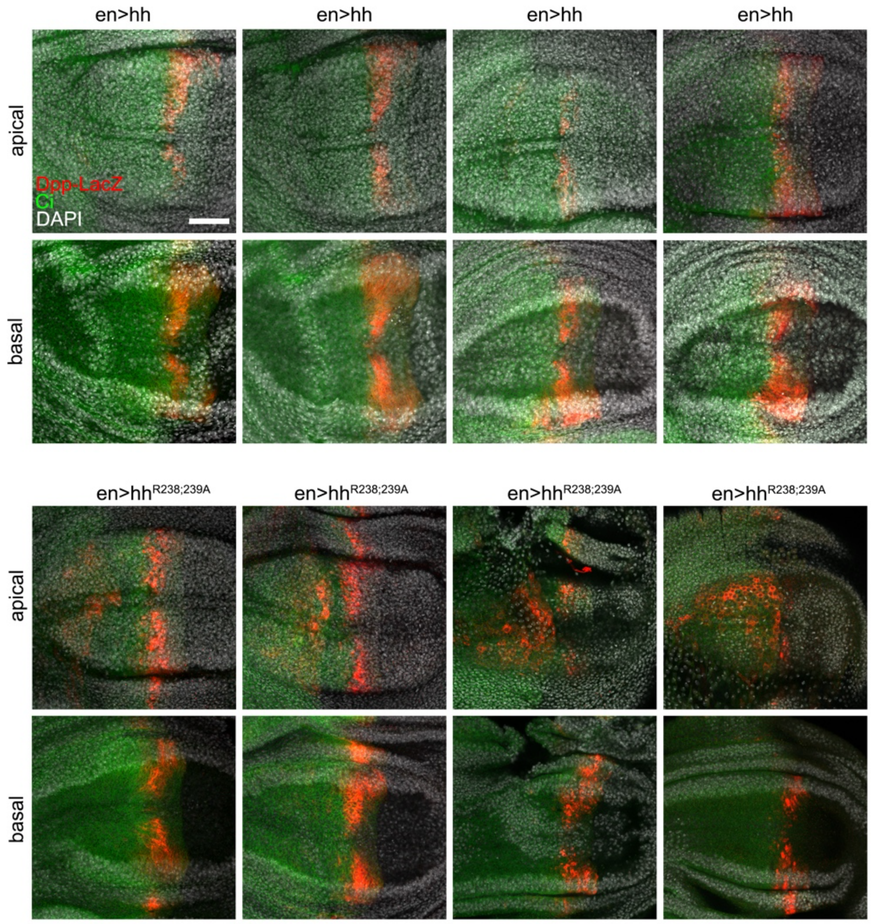

Figure supplement 5. En-Gal4-expressed Hh$^{R238;239A}$ signals to the peripodial membrane. Dpp-LacZ expression is normally restricted to a stripe in the anterior compartment adjacent to the A/P border (top panel). Four wing discs of each genotype are shown. Hh$^{R238;239A}$ overexpression in the producing compartment under the same en control, however, consistently induced ectopic Dpp-LacZ expression in the anterior peripodial membrane (bottom panel). En-controlled expression of Hh never resulted in apical Dpp-LacZ expression in peripodial membranes (top panel). Wing discs are oriented such that posterior is right. Anti-β-Gal staining visualized Dpp-LacZ (red). Anti-Ci-antibodies indicate the Hh-receiving (anterior) compartment (green). DAPI served as a nuclear counterstain (white). Scale bar: 50μm.

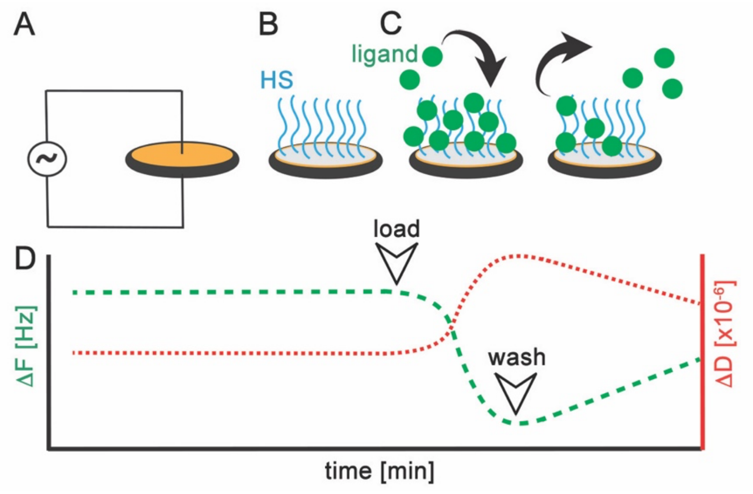

Figure supplement 6. Quartz crystal microbalance with dissipation monitoring (QCM-D). (A) The core of the QCM technology is an oscillating quartz crystal sensor disc with a resonance frequency related to the mass of the disk. This allows the real-time detection of nanoscale mass changes on the sensor surface by monitoring changes of the resonance frequency (ΔF). **(B)** Interaction surfaces were built on heparin (blue) linked to fluid supported lipid bilayers (SLBs, grey) as a proxy for cell-surface linked HS. Like cell-surface HS attached to GPI-linked glypicans, SLB-linked heparin can rotate freely and move laterally on the sensor surface. **(C)** Adsorption of molecules to the surface (green) decrease F, and mass decrease during washing will increase F (shown in **D**). QCM-D measures an additional parameter, the change in energy dissipation D, that is particularly useful in the study of viscoelastic properties of the layer. An increased ΔD during protein binding to the functionalized surface (as shown) correlates with a softer layer and a decreased ΔD would indicate layer stiffening, for example via cross-linking of heparin chains by the bound molecules.

Kusche-Gullberg, M., K. Nybakken, N. Perrimon, and U. Lindahl. 2012. Drosophila heparan sulfate, a novel design. *J Biol Chem*. 287:21950-21956.

2.2 Supplementary Tables

Table S1: Summary of Gel filtration, heparin-, and heparan sulfate elution profiles of Hh/Shh and all Hh/Shh variants employed in this study.

Replaced amino acids (with A)	*wash*	*elution [salt]*	*monomers per total Hh/Shh*
**Heparin FPLC of Shh (mouse)**			**Gel filtration**
Shh	*none*	*1.2 M- 1.5 M*	*1%*
$Shh^{K34A;R38A}$	none	1.2 M- 1.5 M	0%
$Shh^{K33A;R35A;K39A}$	*none*	*1.2 M- 1.5 M*	*0%*
Shh^{K46A}	*none*	*1.2 M- 1.5 M*	*0%*
Shh^{K88A}	*none*	*1.2 M- 1.5 M*	*0%*
Shh^{K179A}	*none*	*1.2 M- 1.5 M*	*0%*
$Shh^{R102A;K104A}$	*none*	*1.2 M- 1.5 M*	*0%*
$Shh^{K46A;K88A}$	*none*	*1.2 M- 1.5 M*	*0%*
$Shh^{K46A;R102A;K104A}$	none	1.2 M- 1.5 M	0%
$Shh^{K179A;K46A;R102A;K104A}$	none	1.2 M- 1.5 M	0%

$Shh^{K46A;K88A;R102A;K104A}$	none	1.2 M- 1.5 M	0%
$Shh^{K179A;K46A;K88A;R102A;K104A}$	none	1.05 M- 1.5 M	7%
$Shh^{K33A;R35A;K39A;K46A}$	none	1.2 M- 1.5 M	0%
$Shh^{K179A;K33A;R35A;K39A}$	*none*	*1.2 M- 1.5 M*	*0%*
$Shh^{K33A;R35A;K39A;K46A;K88A;R102A;K104A}$	none	0.9 M- 1.5 M	1%
$Shh^{K179A;K88A;R124A;R154A;R156A}$	none	1.2 M- 1.5 M	0%
$Shh^{K179A;K33A;R35A;K39A;K46A;K88A;R102A;K104A}$	~50%	0.9 M- 1.5 M	0%
$Shh^{K179A;K33A;R35A;K39A;K46A;R102A;K104A}$	~40%	1.05 M- 1.5 M	0%
**Heparin FPLC of Hh (fly)**			
Hh	*none*	*0.9 M- 1.3 M*	*0%*
$Hh^{R93A;R95A;R97A}$ (=$Shh^{K33A;R35A;K39A}$)	*none*	*0.9 M- 1.2 M*	*0%*
Hh^{K105A} (=Shh^{K46A})	*none*	*0.9 M- 1.3 M*	*0%*
Hh^{R147A} (=Shh^{K88A})	*none*	*0.9 M- 1.3 M*	*0%*
$Hh^{R238A;R239A}$ (=Shh^{K179A})	*none*	*0.8 M- 1.1 M*	*0%*
$Hh^{R161A;K163A}$ (=$Shh^{R102A;K104A}$)	*none*	*1.0 M*	*0%*
$Hh^{K105A;R147A}$	*~10%*	*0.9 M- 1.1 M*	*0%*
$Hh^{R93A;R95A;R97A;R213A}$ (=Shh R154)	none	0.8 M- 1.2 M	3%
$Hh^{R93A;R95A;R97A;R238A;R239A}$	*~20%*	*0.7 M- 1.1 M*	*0%*
$Hh^{R93A;R95A;R97A;R238A;R239A;K105A;R213A}$	~20%	0.7 M- 1.0 M	0%
$Hh^{R93A;R95A;R97A;R238A;R239A;R147A;R213A}$	~40%	0.7 M- 1.0 M	0%
$Hh^{R93A;R95A;R97A;R238A;R239A;R213A}$	~20%	0.7 M- 1.1 M	3%
$Hh^{R93A;R95A;R97A;R238A;R239A;K105A;R147A;R213A}$	~40%	0.7 M- 1.0 M	0%
**HS FPLC of Shh (mouse)**			
Shh	*~40%*	*0.9 M- 1.5 M*	
$Shh^{K34A;R38A}$	~80%	0.9 M- 1.35 M	
$Shh^{K33A;R35A;K39A}$	*~100%*	*none*	
Shh^{K46A}	*~20%*	*0.9 M- 1.5 M*	
Shh^{K88A}	*~40%*	*0.9 M- 1.5 M*	
Shh^{K179A}	*~80%*	*0.9 M- 1.5 M*	
$Shh^{R102A;K104A}$	*~40%*	*0.7 M- 1.5 M*	
$Shh^{K46A;K88A}$	*~20%*	*0.9 M- 1.5 M*	
$Shh^{K46A;K88A;R102A;K104A}$	~10%	0.75 M- 1.5 M	
$Shh^{K179A;K46A;K88A;R102A;K104A}$	~10%	0.9 M- 1.5 M	
$Shh^{K33A;R35A;K39A;K46A}$	~100%	none	
$Shh^{K179A;K33A;R35A;K39A}$	*~100%*	*none*	
$Shh^{K33A;R35A;K39A;K46A;K88A;R102A;K104A}$	~100%	none	
$Shh^{K179A;K88A;R124A;R154A;R156A}$	~10%	0.9 M- 1.35 M	

Table S2: Source data (Figures 3, 4 and 6).

Genotype	*n*	*mean±s.d.*	*p value*
**Figure 3 *(18°C)***			**hh versus**
GMR>;hh^{bar3}/hh^{AC}	*13*	*155±33*	*p<0.0001*
GMR>hh;hh^{bar3}/hh^{AC}	32	*710±59*	
GMR>$hh^{R238;239A}$;hh^{bar3}/hh^{AC}	*15*	*735±58*	*p=0.47*
GMR>hh^{R147A};hh^{bar3}/hh^{AC}	*15*	*701±50*	*p=0.99*
GMR>hh^{K105A};hh^{bar3}/hh^{AC}	*15*	*840±58*	*p<0.0001*
GMR>$hh^{K105A;R147A}$;hh^{bar3}/hh^{AC}	*8*	*723±24*	*p=0.98*
GMR>$hh^{R161A;1639A}$;hh^{bar3}/hh^{AC}	*16*	*704±36*	*p=0.99*

**Figure 4 (25°C)**			
w^{1118} *(males)*	11	1.136±0.03	p<0.0001
en>hh (males)	11	1.68±0.17	
en>hh$^{R238;239A}$ *(males)*	16	1.39±0.19	p<0.0001
en>hh^{R147A} *(males)*	13	1.73±0.03	p=0.9
en>hh^{K105A} *(males)*	12	1.63±0.17	p=0.91
en>hh$^{K105A;R147A}$ *(males)*	10	1.49±0.15	p=0.016
en>hh$^{R161A;1639A}$ *(males)*	13	1.5±0.15	p=0.015
w^{1118} *(females)*	13	1.11±0.017	p<0.0001
en>hh (females)	13	2.1±0.22	
en>hh$^{R238;239A}$ *(females)*	20	1.48±0.19	p<0.0001
en>hh^{R147A} *(females)*	13	1.68±0.24	p<0.0001
en>hh^{K105A} *(females)*	10	1.79±0.15	p=0.0013
en>hh$^{K105A;R147A}$ *(females)*	11	1.47±0.26	p<0.0001
en>hh$^{R161A;1639A}$ *(females)*	14	1.6±0.09	p<0.0001
**Figure 4 (27°C)**			
w^{1118} *(males)*	11	1.14±0.03	p<0.0001
en>hh (males)	10	2.05±0.3	
en>hh$^{R238;239A}$ *(males)*	10	1.36±0.19	p<0.0001
en>hh^{R147A} *(males)*	4	1.87±0.1	p=0.3616
en>hh^{K105A} *(males)*	11	1.97±0.21	p=0.8
en>hh$^{K105A;R147A}$ *(males)*	10	1.63±0.17	p<0.0001
en>hh$^{R161A;1639A}$ *(males)*	10	1.66±0.09	p<0.0001
w^{1118} *(females)*	13	1.11±0.02	p<0.0001
en>hh (females)	10	2.12±0.14	
en>hh$^{R238;239A}$ *(females)*	10	1.5±0.05	p<0.0001
en>hh^{R147A} *(females)*	4	2.1±0.05	p=0.99
en>hh^{K105A} *(females)*	11	2.1±0.25	p=0.95
en>hh$^{K105A;R147A}$ *(females)*	10	1.75±0.17	p<0.0001
en>hh$^{R161A;1639A}$ *(females)*	10	1.75±0.09	p<0.0001
**Figure 6**			
Shh	4	4.9±2.1	
Shh^{K179A}	3	10.9±1.7	p=0.0115
**Figure 7**			
en>GFP (L3-L4/L2-L3)	15	1.07±0.04	
en>hh$^{R238;239A}$ *(L3-L4/L2-L3)*	22	1.16±0.23	p=0.28 (en>GFP)
en>hh (L3-L4/L2-L3)	24	1.98±0.17	p<0.0001 (en>GFP, hh$^{R238;239A}$)
en>GFP (L3-L4)	15	52519±3119	
en>hh$^{R238;239A}$ *(L3-L4)*	9	58980±10540	p=0.12 (en>GFP)
en>hh (L3-L4)	7	69078±6291	p<0.0001 (en>GFP)
en>GFP (L2-L3)	15	49215±2772	
en>hh$^{R238;239A}$ *(L2-L3)*	9	51670±7867	p=0.92 (en>GFP)
en>hh (L2-L3)	7	34171±3696	p<0.0001 (en>GFP)
